# Machine Learning Developed a Programmed Cell Death Signature for Predicting Prognosis, Ecosystem, and Drug Sensitivity in Ovarian Cancer

**DOI:** 10.1155/2023/7365503

**Published:** 2023-10-11

**Authors:** Le Wang, Xi Chen, Lei Song, Hua Zou

**Affiliations:** ^1^Department of Blood Transfusion, The Second Affiliated Hospital of Nanchang University, Nanchang 330000, China; ^2^Department of Emergency, The Second Affiliated Hospital of Nanchang University, Nanchang 330000, China; ^3^Department of General Practice, The Second Affiliated Hospital of Nanchang University, Nanchang 330000, China; ^4^Department of Organ Transplantation, The Second Affiliated Hospital of Nanchang University, Nanchang 330000, China

## Abstract

**Background:**

Ovarian cancer (OC) is the leading cause of gynecological cancer death and the fifth most common cause of cancer-related death in women in America. Programmed cell death played a vital role in tumor progression and immunotherapy response in cancer.

**Methods:**

The prognostic cell death signature (CDS) was constructed with an integrative machine learning procedure, including 10 methods, using TCGA, GSE14764, GSE26193, GSE26712, GSE63885, and GSE140082 datasets. Several methods and single-cell analysis were used to explore the correlation between CDS and the ecosystem and therapy response of OC patients.

**Results:**

The prognostic CDS constructed by the combination of StepCox (*n* = both) + Enet (alpha = 0.2) acted as an independent risk factor for the overall survival (OS) of OC patients and showed stable and powerful performance in predicting the OS rate of OC patients. Compared with tumor grade, clinical stage, and many developed signatures, the CDS had a higher C-index. OC patients with low CDS score had a higher level of CD8+ cytotoxic T, B cell, and M1-like macrophage, representing a related immunoactivated ecosystem. A low CDS score indicated a higher PD1 and CTLA4 immunophenoscore, higher tumor mutation burden score, lower tumor immune dysfunction and exclusion score, and lower tumor escape score in OC, demonstrating a better immunotherapy response. OC patients with high CDS score had a higher gene set score of cancer-related hallmarks, including angiogenesis, epithelial–mesenchymal transition, hypoxia, glycolysis, and notch signaling.

**Conclusion:**

The current study constructed a novel CDS for OC, which could serve as an indicator for predicting the prognosis, ecosystem, and immunotherapy benefits of OC patients.

## 1. Introduction

Ovarian cancer (OC) is the leading cause of gynecological cancer death and the fifth most common cause of cancer-related death in women in America [1]. A total of 19,880 cases are estimated to be initially diagnosed with OC, and 12,810 patients die from this malignancy in America in 2022 [2]. Despite many management approaches that have been used for the therapy of OC patients, including surgery, chemotherapy, and endocrine therapy, the clinical outcomes of OC patients are still poor, with the 5-year survival rate for OC patients less than 50% [1]. In addition to the tumor-node-metastasis staging system, there are few clinical markers used to predict the prognosis of OC patients. High recurrence and drug resistance remained the main reasons leading to the poor clinical outcomes for OC patients [3]. Drug resistance and tumor relapse are the main reasons for the treatment failure [4]. Due to the lack of typical clinical symptoms in the early stage, many OC patients have advanced disease or distant metastases by the time OC is initially diagnosed. A recent study showed that immunotherapy could be a promising modality for many malignancies, especially for the advanced malignancies [5]. However, the evidences about OC response to immunotherapy and the biomarkers for predicting the immunotherapy response are limited.

According to the triggering mechanism, cell death could be divided into accident cell death and programmed cell death (PCD) [6]. As far as we know, PCD could be divided into 15 subtype patterns, including pyroptosis, ferroptosis, necroptosis, autophagy, immunologic cell death, entotic cell death genes, cuproptosis, parthanatos, lysosome-dependent cell death, intrinsic apoptosis, extrinsic apoptosis, necrosis, anoikis, apoptosis-like morphology and necrosis-like morphology [6–8]. Pyroptosis could regulate tumor cell proliferation, metastasis, and affect immune response [9]. Previous study showed that cuproptosis could regulate the microenvironment and affect prognosis in several types of cancer [10]. Increasing evidences highlight the vital role of ferroptosis in reversing drug resistance [11]. As a key player in cellular and body metabolism, autophagy is associated with the progression and prognosis of cancer [12]. As an emerging hallmark in health and diseases, anoikis plays a vital role in tumor progression and drug resistance [13]. Due to the vital role of these PCD in cancer, a comprehensive understanding of the prevalence of PCD-related genes in OC and their correlation to patient's prognosis, ecosystem, and therapeutic response may yield many interesting findings.

In our study, we developed a 21 gene-based cell death signature (CDS) for predicting the prognosis of OC patients in the TCGA cohort. The CDS was verified using five testing cohorts, including GSE14764, GSE26193, GSE26712, GSE63885, and GSE140082 cohort. We then explored the correlation between CDS and the prognosis, immune infiltration, ecosystem, and signaling pathway in OC, offering insights into prognosis prediction and immune landscape in OC.

## 2. Materials and Methods

### 2.1. Datasets Sources

**Figure 1:**
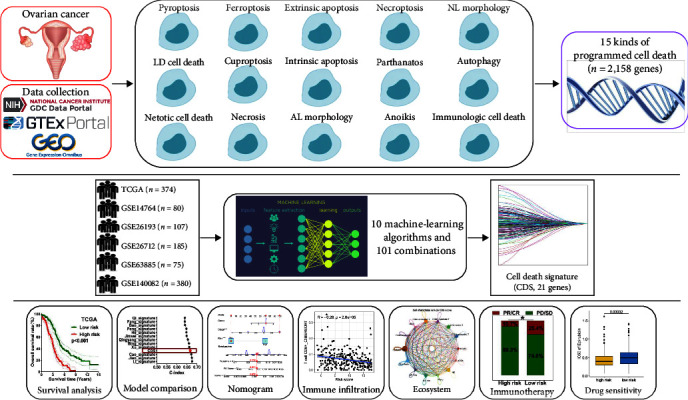
Workflow of our study.

Figure 1 shows the workflow of our study. Related genes of these 15 PCD patterns mentioned above were collated from MSigDB (http://software.broadinstitute.org/gsea/msigdb/index.jsp), Kyoto Encyclopedia of Genes and Genomes, review articles, and manual collection of gene sets from Gene cards website (https://www.genecards.org/) [14, 15] (*Supplementary 1*). Bulk RNA-seq data of OC cases (*n* = 374) and normal ovarian cases (*n* = 64) were obtained from the TCGA database (https://portal.gdc.cancer.gov/repository) and GTEx database (https://xenabrowser.net/datapages/), respectively. We also used five GEO datasets to verify the prognostic signature, including GSE14764 (*n* = 80), GSE26193 (*n* = 107), GSE26712 (*n* = 185), GSE63885 (*n* = 75), and GSE140082 (*n* = 380). Selection criteria of OC cases included the following: (1) histologically diagnosed with ovarian serous cystadenocarcinoma; (2) complete and valid information about age, tumor grade, stage, and overall survival (OS). Exclusion criteria of cases included the following: (1) metastatic OC, (2) accompanied by other malignancies; and (3) no adjuvant therapy before operation. From the GSE184880 dataset, we obtained single-cell expression data of seven OC tissues. IMvigor210 dataset (*n* = 298) and GSE91061 dataset (*n* = 98), containing clinical information about the patients treated with anti-PD-L1 and anti-CTLA4 agents, were used to evaluate the performance of CDS in predicting immunotherapy benefits. Responders were defined as those patients with partial response (PR) and complete response (CR). Nonresponders were defined as patients with progressive disease (PD) and stable disease (SD).

### 2.2. Machine-Learning Algorithms Developed a Prognostic CDS

To obtain the differentially expressed genes (DEGs) in OC among PCD-related genes, we used the “limma” package using |LogFC| ≥ 1 as the cutoff. After obtaining potential prognostic biomarkers with univariate Cox analysis, we then summited these prognostic biomarkers to integrative analysis procedure with 10 machine-learning algorithms, including random survival forest, elastic network (Enet), Lasso, Ridge, stepwise Cox, CoxBoost, partial least squares regression for Cox (plsRcox), supervised principal components (SuperPC), generalized boosted regression modeling, and survival support vector machine, with which we could develop an accurate and stable prognostic CDS. The signature generation procedure was as follows: (1) Prognostic biomarkers were generated using univariate Cox regression in the TCGA dataset; (2) then, 101 algorithm combinations were performed on the prognostic signature to fit prediction models based on the leave-one-out cross-validation framework in the TCGA dataset; (3) all models were detected in five GEO cohorts (GSE14764, GSE26193, GSE26712, GSE63885, and GSE140082); (4) for each model, the Harrell's concordance index (C-index) was calculated across all TCGA and GEO datasets, and the model with the highest average C-index was considered optimal. Similar machine learning algorithms could be seen in previous studies [16–18]. The parameter tuning details about the R scripts in our study are available on the GitHub website (https://github.com/Zaoqu-Liu/IRLS).

### 2.3. Evaluation of the Performance of CDS

Based on the expression of genes in CDS and their corresponding coefficients, we then calculated the CDS score of each OC case. To separate OC cases into low and high CDS score groups, we applied the “surv_cutpoint” function of the R package “survminer” to determine the cutoff. Using the “pROC” package, we then generated time C-index curves. C-index curves were used to compare the performance of CDS in predicting the clinical outcome with 54 prognostic signatures (mRNA and lncRNA-related signatures, *Supplementary 2*) that have been developed for OC. By searching “prognostic model AND ovarian cancer” or “prognostic signature AND ovarian cancer” in Pubmed (https://pubmed.ncbi.nlm.nih.gov/) on February 1, 2023, we obtained a total of 540 signatures that have developed for OC. We used Excel to generate 54 random numbers from 1 to 540, and these 54 random numbers corresponding to the items were selected for further comparison with our prognostic signature. To identify the risk factor for the prognosis of OC, we then conducted univariate and multivariate Cox analysis. Using “nomogramEx” R package, we then developed a predicted nomogram considering CDS, tumor grade, and tumor stage. When the calibration curve is considered a perfectly calibrated model, the predicted value will fall on the diagonal 45° in the figure.

### 2.4. Immune Infiltration Analysis

The correlation between CDS score and immune cells was analyzed with immunedeconv, an R package integrating seven state-of-the-art algorithms, including CIBERSORT, MCPcounter, QUANTISEQ, XCELL, CIBERSORT-ABS, TIMER, and EPIC [19]. By using “estimate” R package [20], we then calculated the immune and ESTIMATE score of each OC case. Single sample gene set enrichment analyses were used to explore the score of immune cells and related functions of each OC case. The normalized enrichment score (|NES| > 1), nominal *p*-value < 0.05 (NOM *p*-value), and false discovery rate-adjusted *q*-value < 0.25 were considered as significant pathway enrichment.

### 2.5. scRNA-Seq Analysis

scRNA-seq data were processed with the Seurat R package (version 4.0) [21]. Those genes detected in more than three cells, cells with more than 200 detected genes, or cells with a mitochondrial proportion of less than 20% would be selected for further analysis. The top 2,000 highly variable genes of each sample were normalized using the ScaleData function based on variance stabilization transformation. The dimensionality of the principal component analysis was reduced using the RunPCA function. We chose dim = 30 and clustered the cells into different cell groups using “FindNeighbors” and “FindClusters” functions. The resolution was 0.5. T-SNE (t-distributed stochastic neighbor embedding), a nonlinear dimension reduction method in Seurat, was applied to map high dimensional cellular data into a 2D space, grouping cells with similar expression patterns and separating those with different expression patterns. The CDS score of each cell was calculated using the AddModuleScore function. Based on the ligand-receptor information, we used the single-cell gene expression matrix to unravel the communication between different cell subtypes, which was contained in CellChat software with default parameters, modeling the communication probability and identifying significant communications.

### 2.6. Drug Sensitivity and Gene Set Enrichment Analyses

To evaluate the role of CDS in predicting the immunotherapy benefits, we then applied the tumor immune dysfunction and exclusion (TIDE) score, immunophenoscore (IPS), tumor mutation burden (TMB) score, and tumor escape score. IPS of OC cases were obtained from the Cancer Immunome Atlas (TCIA, https://tcia.at/home). And the TIDE score and T-cell exclusion scores of OC cases were downloaded from TIDE (http://tide.dfci.harvard.edu). By using the oncoPredict R package, we then calculated The IC_50_ of drugs in each OC case based on the data of Genomics of Drug Sensitivity in Cancer (https://www.cancerrxgene.org/). The sensitivities of these drugs were reflected by comparing the IC_50_ values in patients with high- and low-CDS scores. A higher IC_50_ value indicated lower sensitivity. The Hallmark gene set was downloaded from the Molecular Signatures Database (MSigDB). The gene sets “h.all.v7.4.symbols.gmt” were chosen as the reference gene set. Using the R packages clustersProfiler, enrichplot, and ggplot2, we performed ssGSEA to improve our understanding of CDS-related functions and pathways.

### 2.7. Statistical Analysis

Statistical analyses were performed with R software (version 4.2.1). DEGs in OC were extracted by the limma package. The *χ*^2^ test was applied to compare categorical variables, and the differences between continuous variables were evaluated with the Wilcoxon rank-sum test or Student's *t*-test. The optimal cutoff value was determined by the survminer package. Pearson's or Spearman's rank correlation analysis was conducted to analyze the correlations between two continuous variables. The survival package was applied to conduct Cox regression and Kaplan–Meier analyses. The CompareC package was used to calculate the C-indices of different variables. The predictive value of binary categorical variables was determined by the receiver operating characteristic (ROC) curve with the pROC package. The time-dependent area under the ROC curve (AUC) was calculated by the time ROC package. The proportional hazards assumption for the prediction models was verified using Schoenfeld residuals with the null hypothesis of a slope of zero when scaled Schoenfeld residuals were regressed over time. Failure to reject this hypothesis was considered a verification of the proportional hazard assumption. The fit was assessed using Cox-Snell residuals plotted against the Nelson–Aalen cumulative hazard estimate.

## 3. Results

### 3.1. The Relationship of PCD-Related Genes with the Prognosis of OC

Among 2,158 PCD-related genes, we obtained 1,275 DEGs in OC with |LogFC| ≥ 1 and *p*-value > 0.05 as the cutoff (*Supplementary 3*). Among these genes, a total of 38 genes were significantly associated with the prognosis of OC (*Supplementary 3*). SIRT5, SEC22B, CASP2, IER3IP1, SSBP1, SYNCRIP, TPM3, GBP1, CALM1, STAT1, DNAJA1, and MIF were independent predictors of a favorable OS in OC. High expression of RPL23A, FN1, SERPINE1, FLOT2, MMP14, COL5A1, TGM2, PLEKHF1, COL5A2, BRD4, HSPG2, CDKN1B, CXCL12, LRP1, ITGA5, PDK4, PPP1R13L, TIMP3, RB1, FBN1, FPR1, PDGFRA, BRPF1, AGFG1, LIG3, and HIC1 indicated a poor clinical outcome in OC.

### 3.2. Integrative Machine-Learning Algorithms Developed a Prognostic CDS

**Figure 2:**
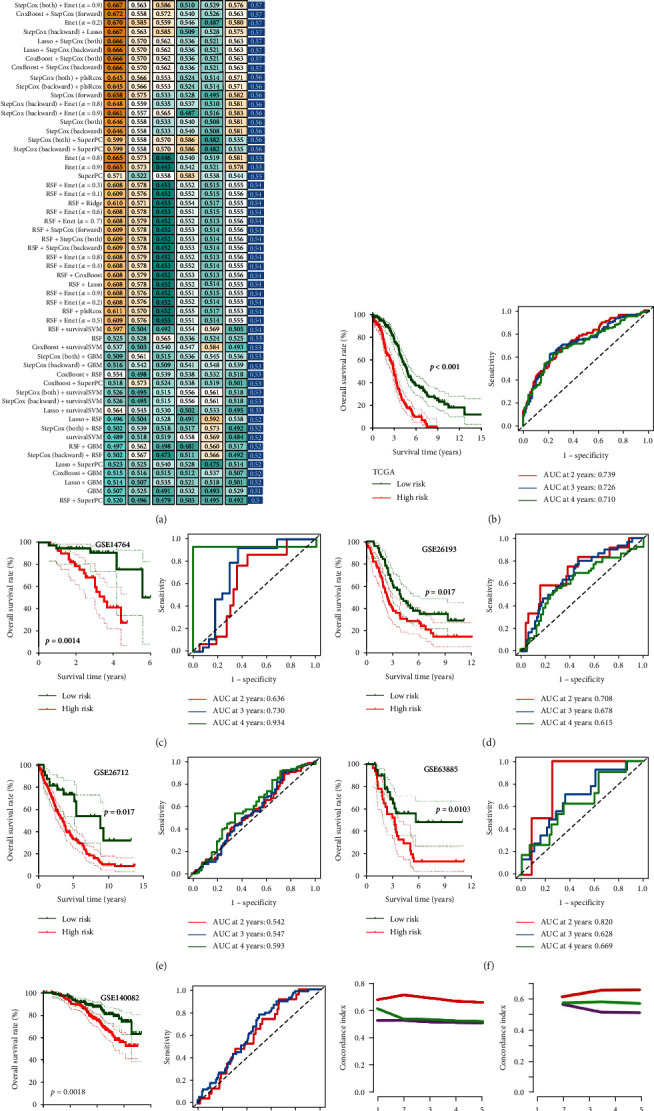
A prognostic CDS developed by machine learning analysis. (a) The C-index of 101 kinds of prognostic models constructed by 10 machine-learning algorithms in training and testing cohort. ((b)–(g)) The survival curve of ovarian cancer patients with different CDS score and their corresponding ROC curves in TCGA, GSE14764, GSE26193, GSE26172, GSE63885, and GSE140082 cohort. ((h)–(l)) The C-index of CDS, tumor grade, and clinical stage in predicting the overall survival rate of OC patients in TCGA, GSE14764, GSE26193, GSE63885, and GSE140082 cohort.

To develop an accurate and stable prognostic CDS, we then submitted these 38 potential prognostic biomarkers to an integrative machine-learning procedure, including 10 methods mentioned above. Finally, we obtained a total of 101 kinds of prognostic models and their C-index of training and testing cohort (Figure 2(a)). The model constructed by StepCox (*n* = both) + Enet (alpha = 0.2) method was considered the optimal prognostic model as they had the highest average C-index of 0.59 (Figure 2(a)). The prognostic CDS was developed by 21 PCD-related genes, and the CDS score of each OC patient was calculated with the formula: risk score = (−0.26736253) × TPM3^exp^ + (−0.15457689) × SYNCRIP^exp^ + (−0.16276132) × CALM1^exp^ + (−0.26067208) × CASP2^exp^ + (−0.16311552) × IER3IP^exp^ + 0.36828974 × AGFG1^exp^ + 0.16153824 × SSBP1^exp^ + 0.28240862 × CDKN1B^exp^ + 0.14662309 × BRPF1^exp^ + 0.09591937 × RB1^exp^ + 0.13519410 × BRD4^exp^ + (−0.14028303) × GBP1^exp^ + (0.12816123) × FLOT2^exp^ + 0.2057739 × PPP1R13L^exp^ + 0.14418808 × FPR1^exp^ + 0.16267570 × TGM2^exp^ + 0.13704459 × LIG3^exp^ + 0.30781520 × COL5A2^exp^ + (−0.28017179) × LRP1^exp^ + (−0.30781684) × SEC22B^exp^ + 0.21714510 × PDK4^exp^. Using the best cutoff value, we then divided into OC cases into high and low CDS (risk) score groups. High-risk scores indicated a poor OS rate in OC in the TCGA cohort (*p* < 0.001, Figure 2(b)), GSE14764 cohort (*p*=0.014, Figure 2(c)), GSE26193 cohort (*p*=0.0017, Figure 2(d)), GSE26712 cohort (*p*=0.0017, Figure 2(e)), GSE63885 cohort (*p*=0.0103, Figure 2(f)) and GSE140082 (*p*=0.0018, Figure 2(g)) cohort, with 2-, 3-, and 4-year AUCs of 0.739, 0.726, and 0.710 in TCGA cohort; 0.636, 0.730, and 0.934 in GSE14764 cohort; 0.708, 0.678, and 0.615 in GSE26193 cohort; 0.542, 0.547, and 0.593 in GSE26712 cohort, 0.820, 0.628, and 0.669 in GSE63885 cohort, 0.603, 0.636, and NA in GSE140082 cohort, respectively. Compared with tumor grade and clinical stage, the C-index of CDS was higher (Figure 2(h)–2(l)) in the training and testing cohort, demonstrating the predictive value of CDS in predicting the OS rate of OC patients was higher than tumor grade and clinical stage. However, we could not evaluate the predictive value of CDS in predicting the OS rate of OC patients in the GSE26712 cohort due to the missing data about tumor grade and clinical stage.

### 3.3. Evaluation of the Performance of CDS

**Figure 3:**
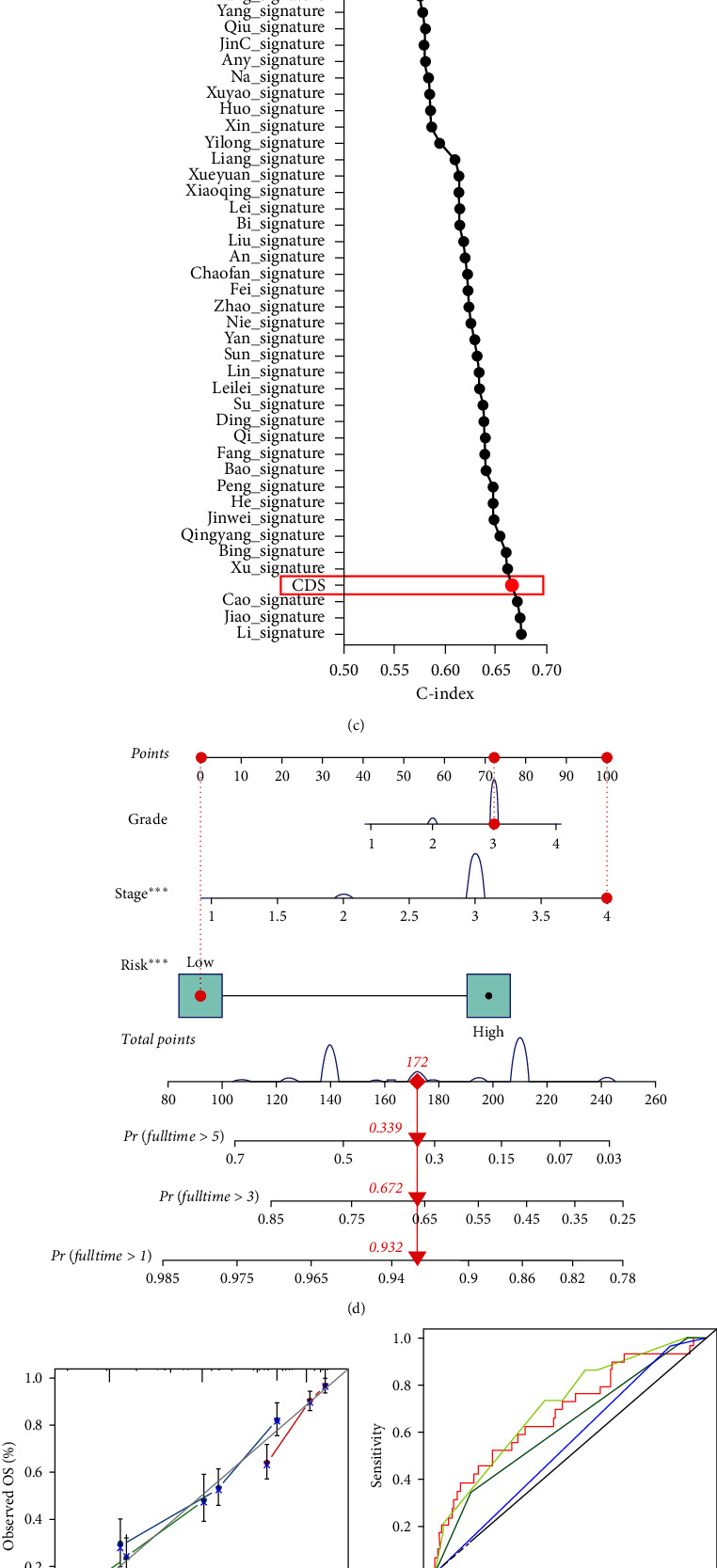
Evaluation of the performance of CDS in predicting the clinical outcome of OC patients. ((a) and (b)) Univariate and multivariate Cox regression analysis considering grade, stage, and CDS in training and testing cohort. (c) C-index of CDS and other 54 established signatures in evaluating the prognosis of OC patients. (d) Predictive nomogram constructed using CDS, grade, and stage. ((e) and (f)) Calibration and ROC curve evaluating the predictive value of nomogram in the overall survival rate of OC patients. (g) DCA demonstrating the good potential of the nomogram for clinical application.

As shown in Figures 3(a) and 3(b), univariate and multivariate Cox regression analysis suggested that CDS-based risk score acted as an independent risk factor for the OS rate of OC in TCGA, GSE14764, GSE26193, GSE63885, and GSE140082 cohort (all *p* < 0.05). Actually, many prognostic signatures have been developed for OC. To compare the predictive value of CDS with other prognostic signatures, we randomly collected 54 prognostic signatures (*Supplementary 2*) and calculated their C-index. Interestingly, the C-index of our CDS was higher than most of these prognostic signatures in the TCGA cohort (Figure 3(c)). Similar results were obtained in the GSE26193 and GSE140082 datasets. In the GSE29193 cohort, the C-index of our CDS was higher than 50 of these prognostic signatures (*Supplementary 4*). And the C-index of our CDS was higher than 48 of these prognostic signatures (*Supplementary 4*). To predict the 1-, 3-, and 5-year OS rate of OC, we then constructed a nomogram considering CDS-based risk score, clinical stage, and tumor grade (Figure 3(d)). Compared with the idea curve, nomogram-based calibration curves had a relative well predictively value in the 1-, 3-, and 5-year OS rate in OC (Figure 3(e)), with the AUC of 0.710 (Figure 3(f)). Moreover, the decision curve analysis (DCA) also suggested that the predictive benefit of nomogram was higher than risk score, tumor grade, and clinical stage (Figure 3(g)).

### 3.4. The Distinct Ecosystem in OC Patients with Different CDS Scores

**Figure 4:**
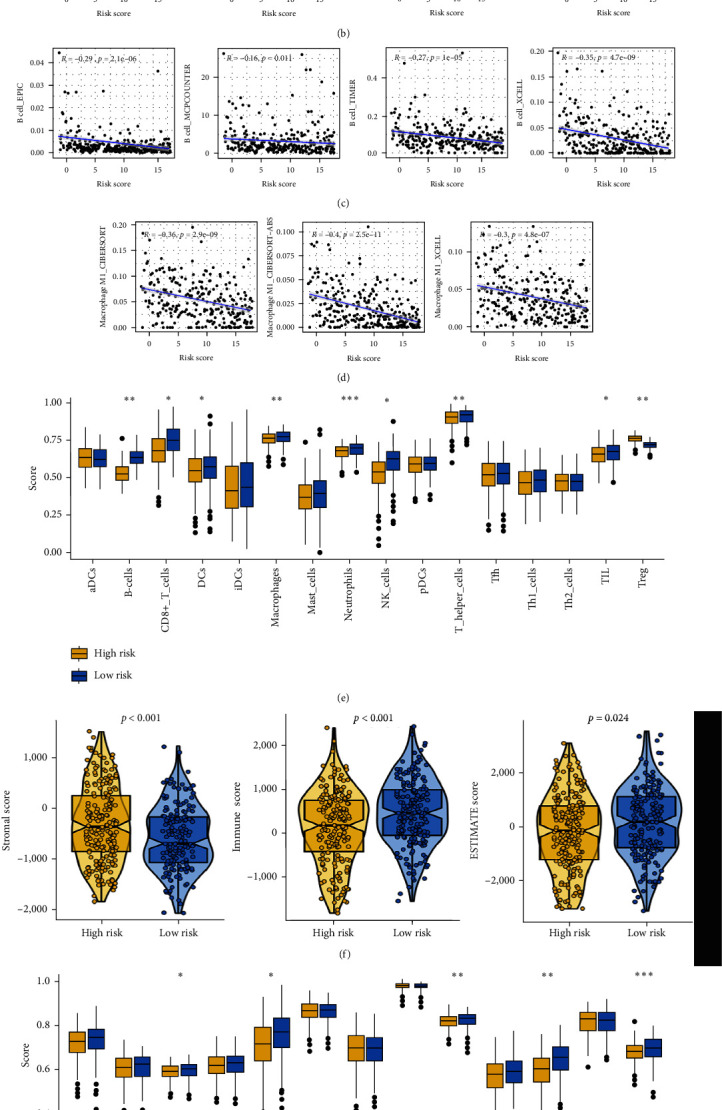
Correlation between immune microenvironment and CDS in OC. (a) The correlation between CDS and the immune cell infiltration is based on several state-of-the-art algorithms. ((b)–(d)) The correlation between CDS and the abundance of CD8^+^ T cells, B cells, and M1-like macrophage. (e) ssGSEA analysis showing the level of immune cells in OC patients with different CDS scores. (f) The stromal score, immune score, and ESTIMAE score in OC patients with different CDS scores. ((g) and (h)) The gene set score correlated with immune-related functions and human leukocyte antigens-related genes in OC patients with different CDS scores.  ^*∗*^*p* < 0.05,  ^*∗∗*^*p* < 0.01,  ^*∗∗∗*^*p* < 0.001.

**Figure 5:**
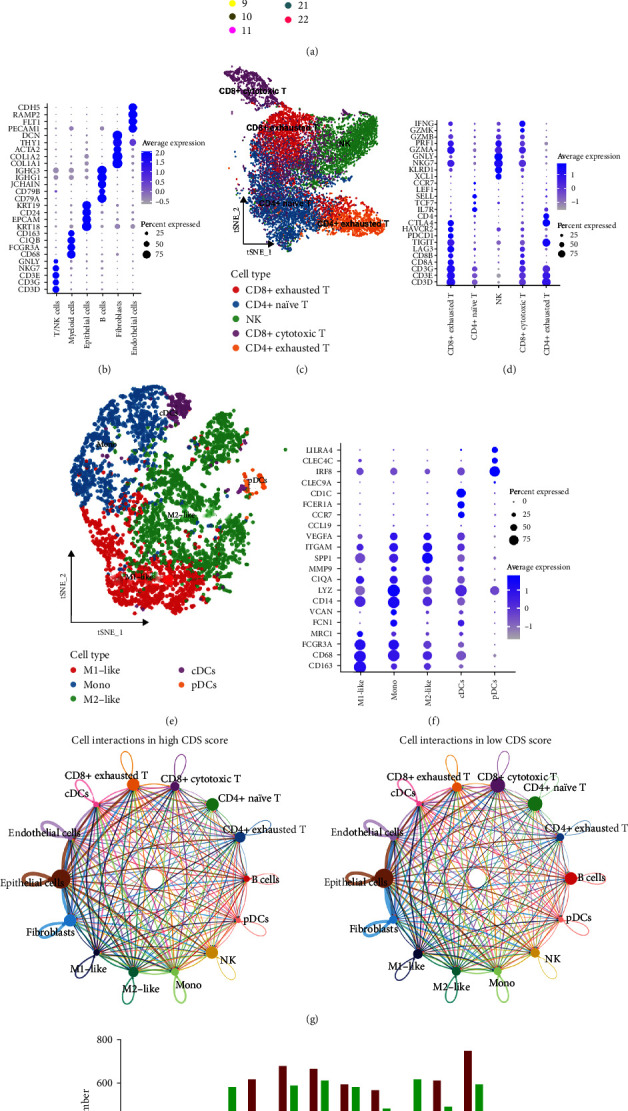
Single-cell analysis revealing the distinct ecosystem in OC patients. (a) tSNE plot of 34 cell clusters and 6 major cell types from 7 OC single-cell samples. (b) Dotplot showing average expression levels of cell marker genes of major cell types. ((c) and (d)) SNE plot of subcell types of T cells and expression pattern of cell markers in dotplot. ((e) and (f)) SNE plot of subcell types of myeloid cells and expression pattern of cell markers in dotplot. (g) CellChat revealing cell–cell communication network via known ligand-receptor pairs in OC samples with different CDS scores. (h) The low CDS-derived B cells, CD8^+^ cytotoxic T, and M1-like macrophage possessed a higher number of ligand-receptor pairs, whereas the CD4^+^ exhauster T and CD8^+^ exhauster T possessed fewer ligand-receptor pairs.

A significant correlation was obtained between CDS-based risk score and the abundance of immune cells (Figure 4(a)). Interestingly, the CDS-based risk score showed a negative correlation with the abundance of immunoactivated cells, such as CD8^+^ T cells, B cells, and macrophage M1 (Figure 4(b)–4(d), all *p* < 0.05). As shown in Figure 4(e), ssGSEA analysis revealed that OC patients with low-risk scores had a higher abundance of immunoactivated cells, including B cells, CD8+ T cells, neutrophils, NK cells, and TIL (all *p* < 0.05). Further analysis revealed that OC patients with low-risk scores had a lower stromal score, higher immune score, and higher ESTIMAE score (Figure 4(f), all *p* < 0.05). A higher gene set score correlated with CC chemokine receptor, cytolytic, parainflammation, and T-cell costimulation was obtained in OC patients with higher CDS scores (Figure 4(g)). Moreover, the level of most of the human leukocyte antigens-related genes was higher in OC patients with low-risk group (Figure 4(h), *p* < 0.05). Based on these findings, we may suggest that the immune environment in OC patients with low and high CDS scores is significantly distinct. As cells exert their functions by interacting with other cells, we then explore the interesting ecosystem between OC patients with different CDS scores. As shown in Figure 5(a), we identified 23 clusters of 7 OC single-cell samples and 6 main types of cells, T/NK cells, myeloid cells, epithelial cells, fibroblasts, B cells, and endothelial cells (Figure 5(a)). And the expression of cell markers is shown in Figure 5(b). Based on the expression pattern of cell markers, T/NK cells could be reclustered into CD8+ cytotoxic T, CD8+ exhausted T, NK, CD4+ exhausted T, and CD4+ naïve T (Figures 5(c) and 5(d)). And myeloid cells could be clustered into M1-like macrophages, M2-like macrophages, monocyte (mono), plasmacytoid DCs (pDCs), and conventional dendritic cells (cDCs) (Figures 5(e) and 5(f)). By using the AddModuleScore function, we then obtained the CDS score of each OC sample and divided them into high and low CDS score groups (Figure 5(g)). To cover the interacting ecosystem of the cells of the high CDS score microenvironment and low CDS score microenvironment, we then used CellChat to construct a cell–cell communication network via known ligand-receptor pairs within these cells in OC samples.  Figure 5(h) shows the cell interactions in high and low CDS score environments. Notably, the low CDS-derived B cells, CD8^+^ cytotoxic T, and M1-like macrophage possessed a higher number of ligand-receptor pairs, whereas the CD4^+^ exhauster T and CD8^+^ exhauster T possessed fewer ligand-receptor pairs (Figure 5(h)).

### 3.5. CDS Could Predict the Therapy Response in OC

**Figure 6:**
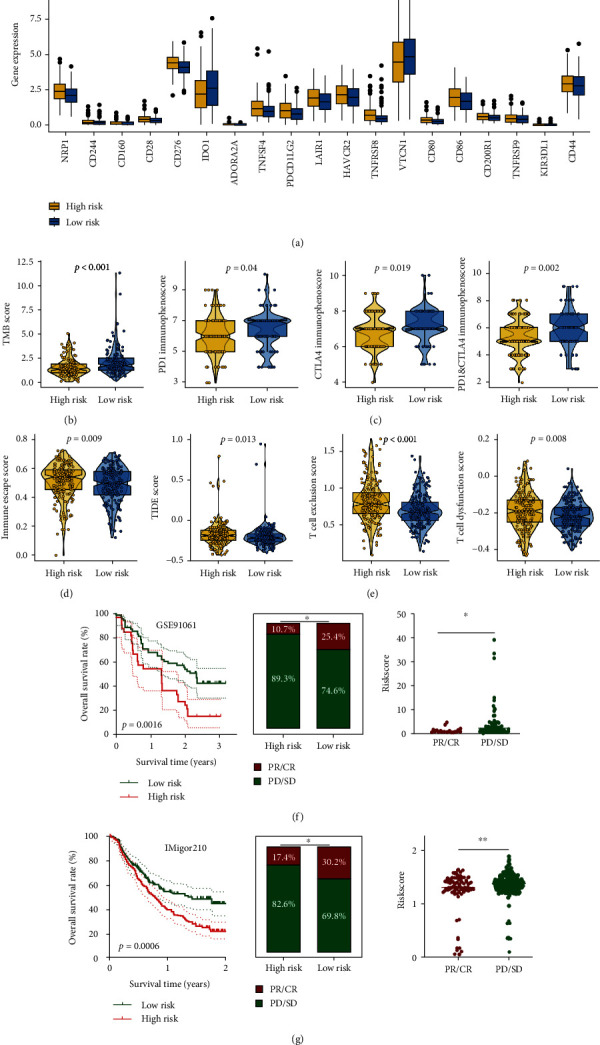
CDS as an indicator for immunotherapy response in OC. (a) The level of immune checkpoints in OC patients with different CDS scores. ((b)–(e)) The TMB score, immunophenoscore, immune escape score, and TIDE, T-cell dysfunction score, T-cell exclusion score in OC patients with high- and low-risk scores ((f) and (g)) The overall rate and immunotherapy response rate in patients with high- and low-risk scores in GSE91061 and IMvigor210 cohort.  ^*∗*^*p* < 0.05,  ^*∗∗*^*p* < 0.01,  ^*∗∗∗*^*p* < 0.001.

**Figure 7:**
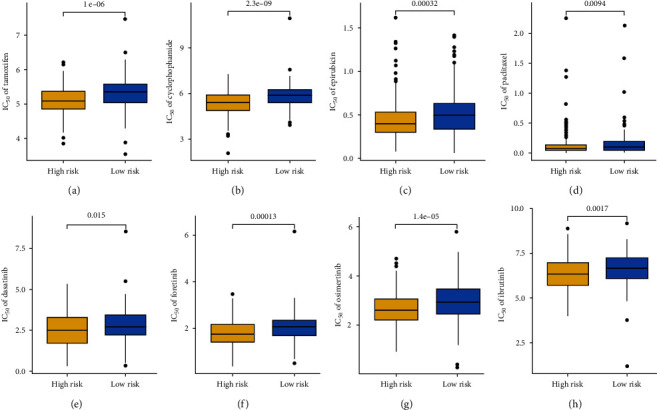
The IC_50_ value of common drugs in OC patients with different CDS scores. OC patients with high-risk scores had a lower IC_50_ value of tamoxifen (a), cyclophosphamide (b), epirubicin (c), paclitaxel (d), dasatinib (e), foretinib (f), osimertinib (g), and ibrutinib (h).

As the ecosystem in OC patients with different risk scores is significantly distinct, the immunotherapy response of OC patients with different risk scores may be different. To verify this, we then applied several approaches to evaluate the predictive value of CDS score in immunotherapy response. Immune checkpoints played a vital role in immune escape from cancer. The data showed that the expression of most of the immune checkpoints was higher in OC patients with high-risk scores (Figure 6(a), all *p* < 0.05). TMB was suggested as a predictive biomarker for predicting the responses to immunotherapy, and a high TMB score indicated a better response to immunotherapy [22]. IPS was a superior predictor of response to anti-CTLA-4 and anti-PD-1 antibodies, and high IPS indicated a better response to immunotherapy [23]. We found that OC patients with low-risk scores had a higher TMB score, higher PD1 immunophenoscore, CTLA4 immunophenoscore, and PD1 and CTLA4 immunophenoscore (Figures 6(b) and 6(c), all *p* < 0.05). A high TIDE score indicates a greater likelihood of immune escape and less effectiveness of ICI treatment [24]. The data showed that OC patients with high-risk scores had a higher immune escape score, TIDE score, T-cell exclusion score, and T-cell dysfunction score (Figures 6(d) and 6(e), all *p* < 0.05). We also used two immunotherapy cohorts to verify our results. In the GSE91061 cohort, patients with high-risk scores had a poor OS rate, and the response rate was significantly higher in patients with low-risk scores (Figure 6(f), *p* < 0.05). Moreover, the response rate in low-risk score group was significantly increased compared with the high-risk score group (Figure 6(f), *p* < 0.05). The risk score in PD/SD patients was significantly higher than that in PR/CR patients (Figure 6(f), *p* < 0.05). Moreover, high-risk scores indicated a poor OS rate in the IMigor210 cohort (Figure 6(g), *p* < 0.05). Compared with patients with high-risk scores, patients with low-risk scores had a higher response rate (Figure 6(g), *p* < 0.05). The risk score in PD/SD patients was significantly higher than that in PR/CR patients in the IMigor210 cohort (Figure 6(g), *p* < 0.01). As the vital role of chemotherapy, targeted therapy, and endocrinotherapy for the treatment of OC. We then explored the IC_50_ value of common drugs in OC patients. As shown in Figure 7(a)–7(h), OC patients with high-risk scores had a lower IC_50_ value of tamoxifen, cyclophosphamide, epirubicin, paclitaxel, dasatinib, foretinib, osimertinib, and ibrutinib, suggesting that OC with high-risk scores may have a better sensitivity to chemotherapy and targeted therapy (all *p* < 0.05).

### 3.6. The Distinct Difference in Cancer-Related Hallmarks in OC Patients with Different CDS Scores

**Figure 8:**
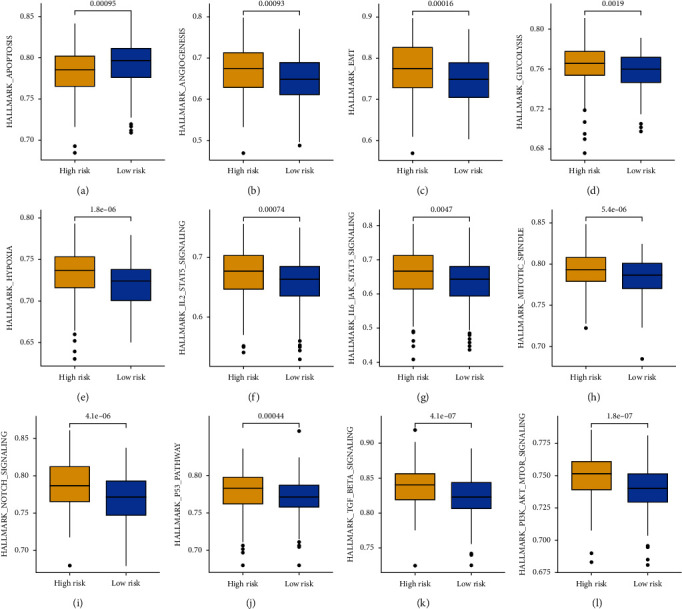
Gene set enrichment analysis in OC patients with different CDS scores. OC patients with high-risk scores had a lower gene set sore correlated with apoptosis (a), higher gene set score correlated with angiogenesis (b), EMT (c), glycolysis (d), hypoxia (e), IL2-STAT5 signaling (f), IL6-JAK-STAT3 signaling (g), mitotic spindle (h), NOTCH signaling (i), P53 pathway (j), TGF-beta signaling (k), and P13K-AKT-mTOR signaling (l).

We finally performed gene set enrichment analysis (GSEA) to explore the potential mechanism mediating the difference of OC patients in clinical outcome, ecosystem, and therapy response. As shown in Figure 8(a)–8(l), OC patients with high-risk score had a lower gene set sore correlated with apoptosis, higher gene set sore correlated with angiogenesis, epithelial–mesenchymal transition (EMT), glycolysis, hypoxia, IL2-STAT5 signaling, IL6-JAK-STAT3 signaling, mitotic spindle, NOTCH signaling, P53 pathway, TGF-Beta signaling, and P13K-AKT-mTOR signaling (all *p* < 0.05).

## 4. Discussion

In our study, we developed a prognostic CDS by the combination of StepCox(*n* = both) + Enet(alpha = 0.2) method in the TCGA dataset. The CDS acted as an independent risk factor for the OS rate in OC and showed stable and powerful performance in predicting patients' OS rate. These findings were also verified in GSE14764, GSE26193, GSE26712, GSE63885, and GSE140082 cohort. Moreover, CDS could serve as an indicator for predicting the ecosystem and immunotherapy benefits of OC patients.

Among these 21 CDS genes (TPM3, SYNCRIP, CALM1, CASP2, IER3IP1, AGFG1, SSBP1, CDKN1B, BRPF1, RB1, BRD4, GBP1, FLOT2, PPP1R13L, FPR1, TGM2, LIG3, COL5A2, LRP1, SEC22B, and PDK4), many have been reported to play a vital role in the development of OC. REDD1 could regulate CASP2-dependent cell death of OC by inhibiting mTOR [25]. BRPF1 played a vital role in the development and progression in OC [26]. A previous study suggested RB1 as an immune-related prognostic biomarker and promising target in OC [27]. BRD4 amplification promoted an oncogenic gene expression program in high-grade serous OC and conferred the sensitivity to bromodomain and extra-terminal motif inhibitors [28]. Serum exosomes LRP1 accelerated the migration of OC patients [29].

Targeting immune checkpoints and activation of antitumor immunity play a vital role in eradicating tumor cells [30]. Immunotherapy offers hope to OC patients with unresectable cancers [31]. However, the evidences about OC response to immunotherapy and biomarkers for predicting the immunotherapy response are limited. A high TIDE score indicates a greater likelihood of immune escape and less effectiveness of ICI treatment [24]. IPS is a superior predictor of response to anti-CTLA-4 and anti-PD-1 antibodies, and high IPS indicates a better response to immunotherapy [23]. In our study, we also explored the role of CDS in predicting the immunotherapy benefit of OC patients. The data showed that OC patients with low CDS scores had a lower immune escape score, lower TIDE, higher TMB, and higher IPS scores, suggesting that OC patients with low CDS scores may benefit more from immunotherapy.

To explore the potential mechanism mediating the difference of OC patients in clinical outcome, ecosystem, and therapy response, we then performed the GSEA analysis. The results showed that OC patients with high-risk scores had a lower gene set sore correlated with apoptosis and a higher gene set score correlated with angiogenesis, EMT, glycolysis, hypoxia, IL2-STAT5 signaling, IL6-JAK-STAT3 signaling, mitotic spindle, NOTCH signaling, P53 pathway, TGF-beta signaling, and P13K-AKT-mTOR signaling. Angiogenesis was correlated with tumor metastasis and as therapeutic targets in OC [32]. OC cells produce chemical resistance by regulating glycolysis, which affects T-cell function [33, 34]. Notch signaling was pivotal for various physiological processes in OC, including immune responses and tumor progression [35]. A previous study showed that hypoxia in the microenvironment could affect the immunotherapy outcome of OC [36].

Many signatures have been developed for predicting the clinical outcome of OC patients. In order to compare the predictive value of our CDI with other signatures. We randomly collected 54 prognostic signatures (*Supplementary 2*) and calculated their C-index. Interestingly, the C-index of our CDS was higher than most of these prognostic signatures in the TCGA, GSE76427, and GSE140082 cohorts, suggesting that the value of our CDI in predicting the clinical outcome of OC patients was better than many prognostic signatures. However, the AUC value of ROC and the C-index of our CDI was not very high, and some of the genes may not be detected by each patient. Therefore, the application of our CDI in predicting the prognosis of OC still needs more clinical OC samples to verify. Moreover, whether the CDI was suitable for other cancers beyond OC need to be further explored.

Some limitations and shortcomings remain in our study. All data were obtained from public databases, and it would be better to validate this prognostic model using clinical data. Moreover, it would be better to explore the mechanism of CDS in the progression of OC. Due to complex models with high-dimensional data, the prognostic signature may fail to generalize to new and unseen data.

## 5. Conclusion

The current study constructed a novel CDS for OC, which could serve as an indicator for predicting the prognosis, ecosystem, and immunotherapy benefits of OC patients.

## Data Availability

The analyzed datasets generated during the study were sourced from the TCGA database (https://portal.gdc.cancer.gov/repository) and the GEO database (https://www.ncbi.nlm.nih.gov/geo).

## Supplementary Materials

Supplementary 1 Information about genes related to different kinds of programmed cell death.

Supplementary 2 Other models have been established for ovarian cancer.

Supplementary 3 Potential biomarkers among PCD-related genes in ovarian cancer.

Supplementary 4 Evaluation of the performance of CDS in predicting the clinical outcome of OC patients.

## References

[1] Armstrong D. K., Alvarez R. D., Backes F. J. (2022). NCCN Guidelines® insights: ovarian cancer, version 3.2022. *Journal of the National Comprehensive Cancer Network*.

[2] Siegel R. L., Miller K. D., Fuchs H. E., Jemal A. (2021). Cancer statistics. *CA: A Cancer Journal for Clinicians*.

[3] Liang L., Yu J., Li J. (2021). Integration of scRNA-Seq and Bulk RNA-Seq to analyse the heterogeneity of ovarian cancer immune cells and establish a molecular risk model. *Frontiers in Oncology*.

[4] Jiang Y., Wang C., Zhou S. (2020). Targeting tumor microenvironment in ovarian cancer: premise and promise. *Biochimica et Biophysica Acta Reviews on Cancer*.

[5] Zhang Y., Zhang Z. (2020). The history and advances in cancer immunotherapy: understanding the characteristics of tumor-infiltrating immune cells and their therapeutic implications. *Cellular & Molecular Immunology*.

[6] Zou Y., Xie J., Zheng S. (2022). Leveraging diverse cell-death patterns to predict the prognosis and drug sensitivity of triple-negative breast cancer patients after surgery. *International Journal of Surgery*.

[7] Müller G. J., Hasseldam H., Rasmussen R. S., Johansen F. F. (2014). Dexamethasone enhances necrosis-like neuronal death in ischemic rat hippocampus involving *μ*-calpain activation. *Experimental Neurology*.

[8] Liu J., Hong M., Li Y., Chen D., Wu Y., Hu Y. (2022). Programmed cell death tunes tumor immunity. *Frontiers in Immunology*.

[9] Lin W., Chen Y., Wu B., Chen Y., Li Z. (2021). Identification of the pyroptosis-related prognostic gene signature and the associated regulation axis in lung adenocarcinoma. *Cell Death Discovery*.

[10] Li C., Xiao Y., Cao H., Chen Y., Li S., Yin F. (2023). Cuproptosis regulates microenvironment and affects prognosis in prostate cancer. *Biological Trace Element Research*.

[11] Zhang C., Liu X., Jin S., Chen Y., Guo R. (2022). Ferroptosis in cancer therapy: a novel approach to reversing drug resistance. *Molecular Cancer*.

[12] Klionsky D. J., Petroni G., Amaravadi R. K. (2021). Autophagy in major human diseases. *The EMBO Journal*.

[13] Wang L., Li C., Wang J. (2022). Transformable ECM deprivation system effectively suppresses renal cell carcinoma by reversing anoikis resistance and increasing chemotherapy sensitivity. *Advanced Materials*.

[14] Tang D., Kang R., Berghe T. V., Vandenabeele P., Kroemer G. (2019). The molecular machinery of regulated cell death. *Cell Research*.

[15] Galluzzi L., Vitale I., Aaronson S. A. (2018). Molecular mechanisms of cell death: recommendations of the Nomenclature Committee on Cell Death 2018. *Cell Death and Differentiation*.

[16] Liu Z., Liu L., Weng S. (2022). Machine learning-based integration develops an immune-derived lncRNA signature for improving outcomes in colorectal cancer. *Nature Communications*.

[17] Liu Z., Guo C. G., Dang Q. (2022). Integrative analysis from multi-center studies identities a consensus machine learning-derived lncRNA signature for stage II/III colorectal cancer. *EBioMedicine*.

[18] Zhang H., Zhang N., Wu W. (2022). Machine learning-based tumor-infiltrating immune cell-associated lncRNAs for predicting prognosis and immunotherapy response in patients with glioblastoma. *Briefings in Bioinformatics*.

[19] Li T., Fu J., Zeng Z. (2020). TIMER2.0 for analysis of tumor-infiltrating immune cells. *Nucleic Acids Research*.

[20] Yoshihara K., Shahmoradgoli M., Martínez E. (2013). Inferring tumour purity and stromal and immune cell admixture from expression data. *Nature Communications*.

[21] Sun Y., Wu L., Zhong Y. (2021). Single-cell landscape of the ecosystem in early-relapse hepatocellular carcinoma. *Cell*.

[22] Liu L., Bai X., Wang J. (2019). Combination of TMB and CNA stratifies prognostic and predictive responses to immunotherapy across metastatic cancer. *Clinical Cancer Research*.

[23] Charoentong P., Finotello F., Angelova M. (2017). Pan-cancer immunogenomic analyses reveal genotype-immunophenotype relationships and predictors of response to checkpoint blockade. *Cell Reports*.

[24] Fu J., Li K., Zhang W. (2020). Large-scale public data reuse to model immunotherapy response and resistance. *Genome Medicine*.

[25] Yang C.-S., Matsuura K., Huang N.-J. (2015). Fatty acid synthase inhibition engages a novel caspase-2 regulatory mechanism to induce ovarian cancer cell death. *Oncogene*.

[26] Zhang J., Li Y., Fan T.-Y. (2022). Identification of bromodomain-containing proteins prognostic value and expression significance based on a genomic landscape analysis of ovarian serous cystadenocarcinoma. *Frontiers in Oncology*.

[27] Xie B., Tan G., Ren J. (2022). RB1 Is an immune-related prognostic biomarker for ovarian cancer. *Frontiers in Oncology*.

[28] Rhyasen G. W., Yao Y., Zhang J. (2018). BRD4 amplification facilitates an oncogenic gene expression program in high-grade serous ovarian cancer and confers sensitivity to BET inhibitors. *PLoS One*.

[29] Zhou W., Ma J., Zhao H. (2023). Serum exosomes from epithelial ovarian cancer patients contain LRP1, which promotes the migration of epithelial ovarian cancer cell. *Molecular & Cellular Proteomics*.

[30] Dai Y., Qiang W., Lin K., Gui Y., Lan X., Wang D. (2021). An immune-related gene signature for predicting survival and immunotherapy efficacy in hepatocellular carcinoma. *Cancer Immunology Immunotherapy*.

[31] Riley R. S., June C. H., Langer R., Mitchell M. J. (2019). Delivery technologies for cancer immunotherapy. *Nature Reviews Drug Discovery*.

[32] He L., Zhu W., Chen Q. (2019). Ovarian cancer cell-secreted exosomal miR-205 promotes metastasis by inducing angiogenesis. *Theranostics*.

[33] Icard P., Shulman S., Farhat D., Steyaert J. M., Alifano M., Lincet H. (2018). How the Warburg effect supports aggressiveness and drug resistance of cancer cells?. *Drug Resistance Updates*.

[34] Zhao E., Maj T., Kryczek I. (2016). Cancer mediates effector T cell dysfunction by targeting microRNAs and EZH2 via glycolysis restriction. *Nature Immunology*.

[35] Canté-Barrett K., Holtzer L., van Ooijen H. (2020). A molecular test for quantifying functional notch signaling pathway activity in human cancer. *Cancers*.

[36] Klemba A., Bodnar L., Was H. (2020). Hypoxia-mediated decrease of ovarian cancer cells reaction to treatment: significance for chemo- and immunotherapies. *International Journal of Molecular Sciences*.

