# Supplementary material for: Machine Learning Developed a Programmed Cell Death Signature for Predicting Prognosis, Ecosystem, and Drug Sensitivity in Ovarian Cancer
Source: Anal Cell Pathol (Amst). 2023 Oct 11;2023:7365503. doi: 10.1155/2023/7365503 (PMC10586435; doi:10.1155/2023/7365503)
Supplement: Supplementary 1 — Information about genes related to different kinds of programmed cell death. [file 7365503.f1.docx]

**Supplementary Table 1. Information about genes related to different kinds of programmed cell death.**

| pyroptosis | parthanatos | necrosis | necrosis like morphology | necroptosis | lysosome dependent cell death | intrinsic apoptosis | immunogenic cell death | ferroptosis | extrinsic apoptosis | Entotic cell death | cuproptosis | autophagy | Apoptosis like morphology | anoikis |
| --- | --- | --- | --- | --- | --- | --- | --- | --- | --- | --- | --- | --- | --- | --- |
| GSDMD  GSDME  NLRP3  CASP1  CASP4  GSDMB  GSDMC  NLRP1  IL1B  GZMB  GSDMA  AIM2  CARD8  CASP8  GZMA  PYCARD  DPP9  CASP5  IL18  ZBP1  CASP3  NLRC4  DPP8  MIR223  CASP6  TRIM24  HMGB1  NAIP  MEFV  DHX9  NLRP9  STAT3  SIRT1  APIP  MALAT1  GAS5  CTSG  NEK7  KCNQ1OT1  TREM2  FOXO3  TP53  MIR30C1  NFE2L2  TXNIP  DDX3X  MIR22  NEAT1  MIR125A  FOXP3  SESN2  ELANE  TNF  GBP1  MEG3  MIR135B  MIR556  FMR1  MIR302A  UBR2  CPTP  GJA1  SNIP1  PRDM1  MIR214  PCSK9  VDR  BRD4  AGER  IKBKE  PKM  KIF23  IFI16  CRTAC1  SEZ6L2  SMIM1  MIR20B  MIR448  TET2  TRAF6  CTSV  UTS2  ZNF532  MIR155HG  MIR155  ZFAS1  MIR527  MLKL  PTPN11  MAPK14  NFKB1  APOE  ANXA1  SDHB  P2RX7  EEF2K  FPR2  HTRA1  PAK2  NDUFA13  CD274  SETD7  FGF21  MIR185  DLX6-AS1  MIR23A  MIR520C  KLF3-AS1  MMP1  IKZF1  BSG  CEBPB  TFAM  PGF  TRIM21  CEP55  CXCL8  NLRX1  SLC16A4  USF2  IL32  PIF1  SLC30A7  CHRFAM7A  MIR21  MIR124-1  MIR200C  MIR195  MIR485  MALT1  TLR2  ICAM1  STK4  GSK3B  PTGS2  MST1  PRF1  PRMT5  VTN  ELAVL1  MPEG1  STING1  MIR204  CDKN2B-AS1  HOTTIP  MIR141  MIR9-1  MIR455  MIR9-2  MIR9-3  MIR497  LINC-ROR  ABL1  EPHA2  HDAC6  PPARG  TLR3  SQSTM1  CDK9  IRF3  UCP1  TREM1  TSLP  ZDHHC1  HNP1  PTEN  DRD2  ADORA1  ADORA2B  ADORA2A  ADORA3  OSM  METTL3  PECAM1  METTL14  TRIM31  MIR25  CAMP  NLRP6  MRE11  FNDC4  FNDC5  MAP3K7  PARP1  USP8  PRKN  GBP5  NR1H2  MKI67  USP47  TCEA3  IL36G  IL36B  TP63  IRF1  IRF2  PRTN3  SERPINB1  IL1A  CHMP4B  DUOX1  APOL1  NLRP7  ANO6  BNIP3  XIST  MIR107  MIR103A1  MIR103A2  FADD  VCAM1  EZH2  DNMT1  DNMT3A  DNMT3B  BAX  CYCS  POLA1  BAK1  CHMP2B  EED  PRIM1  RBBP4  RBBP7  SUZ12  POLA2  CHMP2A  PRIM2  CHMP3  CHMP7  CHMP6  H2AX  CHMP4A  CHMP4C  H3-3A  H3-3B  H3C1  H2AC20  H2AZ1  H2BC21  H4-16  H4C1  H2BC4  H3C2  H3C3  H4C2  H2AC4  H2BC5  H3C12  H3C4  H4C11  H4C3  H4C8  H4C9  H2BC12  H2BC14  H2BC9  H3C10  H3C14  H4C12  H4C14  H4C5  H2AC14  H2AC18  H2AC6  H2AC7  H2AJ  H2AZ2  H2BC1  H2BC10  H2BC11  H2BC13  H2BC3  H2BC6  H3C11  H3C6  H3C7  H3C8  H4C13  H4C4  H4C6  H2AC8  H2BC15  H2BC17  H2BC7  H2BC8  H2BU1  H3C15  H3C13  H4C15  H2AB1  H2AC19  RNF103-CHMP3  LOC102724334  CAPN1  VIM  JUN  RIPK3  MIR139  BRCC3  BHLHE40  BHLHE41  ALK  CSNK1A1  BIRC3  TFAP2A  BIRC2  E2F4  UBE2D2  LY96  GLMN  SUGT1  IRGM  NLRP13  SCAF11  ADAMTS9-AS2  NINJ1  TUBB6  MYD88  TLR8  APAF1  NOS1  NOS2  PKN2  DPEP1  CHMP1A  PYDC2  MUC20  ACE2  EGFR  AKT1  ATF6  CASP9  ORMDL3  POP1  LINC00958  MIR4306  MDM2  BTK  BCL2  YWHAE  ANXA2  HSP90AA1  HSP90AB1  IL1RN  RIPK1  IFIH1  IRAK3  MELK  NEDD4  NFS1  YWHAZ  BECN1  CD14  CHI3L1  STXBP2  TLR9  UBE2D3  GSTO1  RAB5A  TNFSF13B  ASIC1  HUWE1  LRPPRC  PANX1  PDCD6IP  USP24  ATG7  IL13  CDC37  ERP44  IL18BP  TRPM2  VPS4B  ATG3  BST2  IL13RA2  LYST  STXBP3  GPER1  NCR1  VPS28  IL27  CLEC5A  SEC22B  SIGLEC14  CGAS  MIR15A | AIFM1  PARP1  ADPRS  RNF146  NAMPT  GPX4  MAPK8  SQSTM1  CAST  AIMP2  RIPK1  FEN1  ESR1  RAB33A  DIABLO  MCL1  DDB1  CUL4A  TOMM20  OTUD1  DCAF10  PTEN  ESR2 | TNF  TNFRSF1A  COL2A1  TNFRSF1B  TNFSF10  IL6  LTA  IL1B  FAS  IL10  TNFSF11  TRPV4  FASLG  TNFSF13B  TNFRSF10A  IFNG  TNFRSF10B  TRAF2  MT-ATP6  TNFAIP3  TNFRSF14  TNFSF14  CXCL8  CASP8  TNFRSF11B  TNFSF12  TRADD  IL1A  TNFSF4  TNFSF15  TNFSF13  NUP62  TP53  ADAM17  CASP3  RIPK1  CRP  SLC25A19  TNFRSF11A  CD40  TNFRSF18  TNFRSF4  TLR4  TNFSF9  TNFRSF25  CCL2  TNFRSF9  TNFSF18  IL18  TNFAIP6  MAPK8  TNFRSF6B  CD40LG  TNFRSF10C  TNFRSF10D  LTBR  ICAM1  TNFRSF8  FADD  MYLK  TNFSF8  COL1A1  XIAP  ADAR  IL1RN  TNFRSF17  TNFRSF13B  TGFB1  TGFB2  ACTA2  LTB  TRAF3  RIPK3  IL4  TNFRSF19  TRAF1  PTGS2  TNFRSF12A  SMAD3  CD70  ADIPOQ  VCAM1  CSF2  HLA-DRB1  HLA-B  PRKG1  TGFBR1  SLC2A10  CFLAR  ACE  MIF4GD-DT  TNFRSF21  TRAF6  NFKB1  LEP  LOX  MYH11  IL2  ALB  IL4I1  TGFBR2  TGFB3  CYCS  SELE  BIRC3  HMGB1  VEGFA  FLNA  IL1R1  BIRC2  ELN  CASP1  NOTCH1  MAPK1  TRAF5  TNFAIP1  TNFRSF13C  IL17A  SMAD4  TNFAIP2  RELT  CBS  MMP9  FBN1  IFNA1  TERT  HBB  CD27  FOXE3  BAX  JUN  TLR2  MFAP5  NFKBIA  MAPK14  BCL2  PLOD1  CD14  CSF1  MLKL  BGN  CHUK  SMAD2  ESR1  ACTA2-AS1  PARP1  MAT2A  EDAR  IKBKB  JAG1  NOS2  MIR21  MMP1  HMOX1  F3  TNFAIP8L2  SERPINE1  MMP3  RELA  COL3A1  CSF3  MAP3K5  MED12  FBN2  TNFAIP8  INS  COMP  NGFR  NEK9  COL5A1  MEFV  BMP6  EDA  TNFSF12-TNFSF13  GNPTAB  NPM1  COL5A2  NDE1  LOC101448202  LOC113939944  TRAF4  FN1  GBA1  IL1RAPL2  EDA2R  HEY2  F5  GATA4  C1QTNF5  TRAP1  GPT  NOD2  HGF  C1QTNF3  IL2RB  CASP9  HIF1A-AS1  SRFBP1  MYLK-AS1  LINC01389  SMAD6  TIMP1  EP300  ELANE  TANK  SLC12A5-AS1  CCL5  C1QTNF9  IL2RA  STAT3  TINF2  CTLA4  GZMB  ROBO4  PPARG  STAT1  CREBBP  C1QTNF1  STING1  IL12B  H19  ACAN  FGF2  TICAM1  IGF1  MIR155  CERNA3  SOD2  HLA-A  IFNB1  C1QTNF4  OTULIN  IL15  POLG  B2M  LOC100506472  AAT1  ANXA5  TNFAIP8L1  PSMD2  MEG3  AKT1  F2  PTX3  C1QTNF6  IKBKG  TNFAIP8L3  BMP2  CCL3  NPPA  CASP10  CCR6  IRF1  ZFP36  HLA-DQB1  EGF  IL13  SOD1  MAP3K14  ACTA1  NOS3  HSP90AA1  BCL2L1  STAT4  TYMS  IL3  NAGLU  MIR221  PTPN11  REN  MIR146A  BDNF-AS  IRF3  BGLAP  MAPK3  SGCA  CALCA  TERC  RTEL1  RETN  JAK2  MB  MAP3K7  LITAF  ERAP1  NR3C1  MPO  THBD  MMP2  WRAP53  SELP  COL1A2  SLC22A12  HIF1A  C1QTNF2  UMOD  INSR  STEAP4  CTNNB1  APOE  C1QTNF7  CASP4  IFNGR1  APOH  IL23R  RYR1  C1QTNF9B  BAK1  CDKN2B-AS1  LCN2  PTGS1  ACE2  MIR7-3HG  BID  EDN1  TLR9  PTPRC  IL12A  PLA2G4A  CST3  LINC01672  OPTN  MIR210  IL5  MIR130A  STAC3  GHRL  CD4  C1QTNF8  EGFR  FLNC  IL18R1  IL6R  CPT2  CXCL10  CAT  FCGR3A  GSDME  TLR3  PIK3CA  IL11  PRF1  EPO  MIR34A  KITLG  NTRK1  TIMP3  SPP1  C4A  APOL1  PARN  UBC  NHP2  PROC  LINC02605  VDR  IL6ST  RUNX2  HP  BRAF  HLA-C  APOB  SMPD1  RANBP2  CXCR3  PROS1  KRT18  MIF  CASP7  ADAMTS2  DKC1  NOP10  KRAS  NFE2L2  MIR125A  TYK2  MATN3  PIK3C2A  CCL4  HSPA1A  MIR29A  SLC2A1  IRS1  MT-CO1  PSTPIP1  WNT5A  LBP  NLRP3  CCR1  MIR122  TRPS1  MALAT1  CDKN2A  TGFA  POLG2  SOD2-OT1  SELL  ADAMTSL2  VWF  FOS  PRKN  HAVCR1  MYD88  IFNA2  MMP13  MIR214  MTHFR  THSD4  CREB1  MAPKAPK2  MT-ND6  MMP12  TREX1  SH2D1A  PPARA  IL32  BIRC5  MIR132  CCL11  POMC  ADAM10  PIK3CG  CTC1  ADA2  PECAM1  GAPDH  F10  DNM1L  CASP5  SERPINC1  TONSL  MIR146B  SRC  USB1  SP1  OLR1  CASP2  MAP3K1  MYC  IKZF1  MIR222  CTSK  ITGB3  SLC17A5  ELP1  FGFR1  SERPINA1  IGFBP3  RTEL1-TNFRSF6B  DSPP  CD36  CD8A  LIF  ENG  BMP7  IBSP  ACP5  THBS1  AGER  SMAD5-AS1  LPL  APOA1  IRAK1  ITGAM  LOC100287329  ARSB  EGR1  SCARB2  ITGB2  FCGR2A  OGA  MIR181A1  MET  MBL2  MIR223  MMP8  ALOX5  PTH  BPI  RIGI  SAMHD1  MIR23A  EXT1  CDH23  CYLD  RB1  ANGPT1  MECOM  AFP  DVL3  CCL20  SIL1  CDKN3  PRL  FABP1  MIR145  IL33  MMP14  BAG4  IL7 | TP53  AKT1  VIM  MYC  BCL2  KRT5  RAC1  FAS  ABL1  CASP8  IL2RA  AIFM1  LETM1  CASP3  SLC2A1  IFNG  CYCS  TNFRSF1A  PEPD  PGR  FOXP3  PAFAH1B1  KRT20  FASLG  BAX  NLRP3  HIF1A  RIPK1  PARP1  ANXA5  NFKB1  TNFSF10  HTRA2  TKT  CASP9  USF3  TNFSF12  BCL2L1  HSPA5  CAT  GAPDH  AURKA  CASP1  TNFSF15  FGF14  XBP1  CASP2  HMGB1  ENDOG  GP6  MBL2  BNIP3  GSS  SP1  TNFRSF10A  TNFRSF10B  NRIP1  IAPP  CAPN1  BECN1  TNFRSF12A  ENO1  TYMS  PDLIM1  RPAIN  TNFRSF6B  PLA2G7  MYO10  DIABLO  ATG4B  ATG3  MAP1LC3A  PRKAA1 | RIPK1  RIPK3  MLKL  ZBP1  CASP8  TNF  CYLD  ITPK1  IPMK  MAP3K7  CASP6  TRPM7  FADD  PELI1  PGLYRP1  SPATA2  SIRT3  HMGB1  TP53  TNFRSF1A  MEFV  AIM2  TNFAIP3  UCHL1  STING1  TNIP1  SERTAD1  TRAF2  NFKB1  CFLAR  MAPK14  FAS  BIRC2  AIFM1  KLHDC10  SFTPA1  GSK3B  XIAP  MYC  TNIP3  TLR4  GJB1  DNM1L  PTGES3  UVRAG  BRD4  RB1  SIRT2  IKBKB  DAPK1  FKBP1A  MIR425  EZH2  NFE2L2  CD274  CHMP4B  CXCL5  MIR29B1  BCL2  AXL  MERTK  TYRO3  SIRT6  NAT2  SLC39A7  USP22  PANX1  PDCD6IP  FLOT1  FLOT2  IL37  DIABLO  FASN  CDK9  TIMM50  SLC25A37  PPP1R3G  MIR7-1  NFKBIA  AURKC  NGFR  FMR1  GNLY  PARP1  HTRA2  HSPA5  PRKAA2  PRKAA1  PITPNA  METTL3  TRAF5  FNDC4  FNDC5  FASLG  TXN  RALBP1  TP53I3  GSDMD  PRKN  HSP90AA1  STUB1  TNFSF10  CDC37  RELA  TRPC6  SIRT5  MIR21  AURKA  PLK1  TNFRSF10B  MYH9  CASP10  SQSTM1  SOAT1  TNFRSF10A  HGF  SRC  ANXA1  CASP2  FPR1  MIF  BCL2L1  KL  TNFRSF21  BMI1  BBC3  AVEN  ZNF7  PTEN  ESR2  GSK3A  C5  SP1  C9  ATG5  C6  C7  CD74  EGR1  NOX4  RCN1  MKRN1  TNFSF12  UHRF1  AIFM2  CTSB  CD40LG  OGT  BID  IFNB1  ID1  MIR155  TLR3  KIAA1191  CYP3A4  PAK1  SGK1  CDC7  IDO1  SMN1  SMN2  CERK  TRADD  TP63  IL1A  PADI4  MIR214  MIR22  MIR221  MIR101-1  MIR485  MIR101-2  CTSD  CLEC7A  OTULIN  TRIM28  STAT3  PGAM5  HPRT1  FAP  CHL1  TRIM24  TGFBR1  CDKN2A  SNCA  ACVR1B  DDX58  PYGM  TARDBP  CCL2  XRCC1  DPEP1  CHMP1A  UBR2  GPX4  LAMP2  MPRIP  MTOR  BRAF  AFP  ACHE  NFAT5  CXCL1  VIL1  SHARPIN  CBL  MAPK1  MAPK8  TBK1  CTSH  GSN  RARG  BIRC3  CTSL  CTSS  DSTYK  RNF31  TNFRSF25  CHUK  BUB1B  IRAK1  BDNF  EIF2AK3  HSPA8  IFNG  IKBKG  MAPK3  NQO1  TAB2  BECN1  XBP1  TICAM1  RBCK1  SOX17  UBC  BNIP3  HSPA4  TAB1  AGFG1  TAB3  EIF2A  MIB2  TNIP2  H1-5  DIRAS3  C20orf204  EGFR  JAK1  FLT3  IDH1  IDH2  HMOX1  MMP13  ATP2A1  CAD  CASP1  DRD2  EPAS1  G6PD  GJA1  NPM1  TSC2  VCP  VIM  ACTB  AHR  BAX  EEF2  KIF11  PTPN6  RPS19  YWHAE  YWHAG  APC  BAP1  HSP90AB1  HSPD1  KRT18  LEF1  LRP1  MAPKAPK2  NOD2  PIKFYVE  PRDX1  RANBP2  RPL11  SLC16A1  TNNT2  TPM1  AFG3L2  CLTC  HIF1A  HSPA9  KRT1  KRT14  KRT5  KRT8  MYH14  NFS1  NLRP3  PINK1  PPP1CB  PRPS1  PRSS1  RPL5  SLC25A1  SLC25A13  TNFRSF1B  TPM3  TRAF3  TUFM  ARHGEF2  ATF4  CALM1  COPB2  DDX3X  DDX5  EIF4EBP1  FLNC  HNRNPA1  HNRNPA2B1  HSPA1A  IKBKE  IL4  IRF3  LTBP1  MYO5A  MYO6  NUP214  PABPN1  PARK7  PFKL  PKM  RIPK2  TET2  TPM2  TRAF6  TUBA4A  TUBB4A  UBE2D3  UGDH  CALM2  CCT5  FUS  KRT19  LGALS3  MAP1B  OPTN  PKP2  RPL35  RPS10  S100A4  UBE2L3  XRCC6  ACTC1  AHSG  ASIC1  CADM1  EEF1A1  EMD  FLII  GBE1  KRT10  KRT16  KRT6A  LITAF  NDUFA4  NRIP1  PLEC  PNKD  PPM1B  PSMA3  RBMX  RPL15  RPS14  RPS20  RPS3  S100A10  SFPQ  SKP2  SLC25A5  STK38  TCOF1  TCP1  TUBB4B  ULK1  VDAC2  XRCC5  XRN2  AP2S1  BANF1  CALM3  CALU  CAPZA1  CNBP  COPA  CUL4A  HNRNPU  KHDRBS1  KLF6  KRT2  KRT7  KRT9  LGALS1  MYO1C  NCL  NSUN2  PABPC1  PCBP1  PCM1  PRPF8  RNGTT  RPL10A  RPL13  RPL13A  RPL22  RPL26  RPL27  RPL7  RPL7A  RPS12  RPS13  RPS17  RPS24  RPS26  RPS27  RPS6  S100A6  SLC25A10  SLC30A9  TANK  TNFRSF8  TUBA1B  TUBA1C  AGO2  AP2B1  ATAD3A  BCLAF1  CCT3  DNAJA1  DYNLL1  EIF4B  ELAVL1  IGF2BP3  IVNS1ABP  MAP1LC3A  PAWR  PDIA4  PSMC4  PYCARD  RPL12  RPL23A  RPL3  RPL4  RPL9  RPLP2  RPS23  RPS29  SLC25A6  SNRPE  SRSF1  SVIL  TUBB6  CCNT1  CCT6A  CCT8  DDX17  DNAJA2  FIP1L1  HSPA1B  IGF2BP1  IL24  IQSEC1  MVP  MYL6  PDLIM7  RIOK1  RPS16  SF1  SSBP1  SYNCRIP  ADAMTSL4  BAG2  CEP170  CLINT1  COPS2  CPSF3  CPSF6  GOSR1  HNRNPF  HNRNPH1  HNRNPH3  HNRNPL  HNRNPM  HOOK1  KRT86  LARP1  LRRFIP2  MPP1  MRPS12 | TP53  EGFR  ATM  AKT1  TNF  PDCD1  MTOR  IL6  VHL  CD274  DAPK1  RIPK1  ABL1  IL1B  ICAM1  JAK2  STK11  PPARG  SRC  HIF1A  EGF  CFLAR  TGFBR2  PTPN11  CDK4  MCL1  PARP1  TSC2  MAPK14  MITF  PKLR  TSC1  IKBKB  PTGS2  IGF1R  BCL2L11  CBL  CD36  CDK6  GBA  PROM1  HMGB1  FLT1  SQSTM1  SMPD1  CHEK1  GSK3B  YAP1  TFEB  HSPA5  FOXO3  NOTCH3  TRAF2  IL6ST  CTSD  SIRT1  VCP  TRAF6  SHC1  CTSB  STK4  PKM  CD86  ITGA5  CTNND1  PARK7  NLRP3  BECN1  AHR  HDAC6  XRCC5  RIPK3  HSPA8  RPS27A  ANXA2  C9orf72  MAP3K7  IL1R1  HCK  DDR1  VTN  PRKAA1  UBC  IRF3  ARSA  NEDD4  CD1D  LAMP2  KCNB1  IRAK1  CTSK  TAB2  ROR2  GDF15  IFNGR1  TRIM28  CTSL  STUB1  ATG5  CAPN1  LAMP1  RPS6KA3  CUL1  IAPP  RB1CC1  CSNK1A1  PPIA  MCOLN1  SERPINB3  HGS  GAA  RPTOR  CBLB  RAB7A  PEBP1  PHLPP1  APOD  UBB  IFNAR1  RAB5A  CLCN7  DACT1  NUMB  AQP2  CLDN5  MAP1LC3A  VPS33A  TNK2  CSNK1E  NCOA4  ARL8B  ULK1  UBE2D1  SLC1A5  ABL2  MSTN  MAP1LC3B  SNAP23  AGFG1  GABRA1  VPS4B  GABARAPL1  GABARAP  SBF2  UBE2D3  VAMP8  TAB3  VAMP7  ATP13A2  BACE2  MIR33A  CALCOCO2  RNF41  LGALS8  UBE2E1  GABARAPL2  UBE2D2  CBLL1  ULK2  SMCR8  RAB21  ATG13  STX5  MCOLN3  VPS11  MCOLN2  VPS16  SHFL  ILKAP  PDE1C  EI24  PLEKHM1  MREG  CACYBP  FYCO1  GPSM1  ROBO3  ACP2  VPS18  MAEL  SLC2A12  ATG4A  NLRP6  LAMTOR5  VPS41  SIGLEC15  CTSV  SCFD1  STX6  SEC22B  MIR33B  MAP1LC3C  KDM7A  TIFAB | CBLIF  BCL2  CASP8  CASP3  BAX  FAS  XIAP  TP53  CASP9  CFLAR  FASLG  CASP10  AIFM1  TNFSF10  BCL2L1  MCL1  CYCS  BCL2L11  BIRC5  CUBN  CASP7  BOK  TNFRSF10B  TNF  CASP2  CASP6  PRKCD  AKT1  MAPK8  BID  NAIP  CASP1  BIRC2  MIP  PARP1  DIABLO  KRAS  LIM2  PAWR  MAPK1  BAK1  SIVA1  BIRC3  TNFRSF10A  CDKN1A  STAT3  NRAS  BBC3  APAF1  MYC  MAPK14  BAD  MOAP1  RIPK1  API5  MAP3K5  BIRC7  CCAR2  FADD  AEN  CCAR1  AVEN  TNFRSF1A  CASP4  PEA15  ANXA5  JUN  PERP  MDM2  CIAPIN1  BFAR  NOL3  TP53AIP1  AATF  TRIAP1  E2F1  MTOR  TP53BP2  NFKB1  CHEK2  AREL1  DAXX  RAF1  GZMB  EGFR  BCL2L2  DDIAS  CDKN2A  TRAF2  CAAP1  TNFRSF10C  ECSCR  MIR21  TNFRSF10D  MAPK3  FOXO3  CASP14  TP73  ERBB2  BIK  HIF1A  CLU  PTEN  CASP5  LMNA  ATM  HRAS  PYCARD  ELAPOR1  PMAIP1  PTGS2  BECN1  IGF1  APP  CDK1  CD4  DFFA  TGFB1  VEGFA  SEPTIN4  SQSTM1  PML  CCND1  HSPA5  BCL2L10  SOD2  LDB3  HTRA2  MAPK10  BCL2L14  TRADD  IL10  IL2  SIRT1  HINT1  CDK2  ESR1  RB1  ACTA2  IFNG  PPP1R13B  DDIT3  PIK3CG  RELA  MAP2K1  TNFRSF25  PPARG  FLNC  CDKN1B  IGF1R  IL3  AKT2  OPA1  CXCL8  HRK  AATBC  ABL1  NFKBIA  BCL2L13  CTNNB1  IL6  BRCA2  TIA1  TNFSF12  TET2  IL1B  SRC  BRCA1  YWHAQ  NR3C1  MAPK9  AKT3  CBL  PTK2  PCNA  CSE1L  TP63  JAK2  YWHAE  HSP90AA1  XAF1  HDAC9  DFFB  RIPK3  VDAC1  FOS  HSPB1  NFE2L2  DYSF  BDNF  HMGB1  PIK3CA  TCN2  YWHAB  YAP1  BCL2A1  PTPN11  IL4  EGF  CD40  PDCD6  HMOX1  IL5  PRKN  PDCD5  RNF7  F3  TTN  VCP  FAF1  MYH7  GSK3B  TNFRSF1B  TLR4  AR  TXN  FOXO1  NGF  SOD1  CCNA2  SYVN1  CARD8  ABCB1  CHUK  ALB  F10  TRAF1  CAV3  RASGRP1  EP300  MMP9  DSP  CAT  STK4  BRAF  BNIP3  HDAC1  SH3KBP1  TGM2  IGFBP3  CDK5  RARA  IFNA1  BCL10  IKBKB  MIR146A  IRF1  NOS2  YWHAZ  BIRC6  IL2RA  PIK3R1  DAPK1  SP1  BAG3  RIPK2  ENDOG  TNFRSF11B  STAT1  PPP1R13L  C1QBP  DNM1L  MAP2K4  CCNB1  VHL  HIPK2  GSDME  TERT  PRKCA  F8  NR4A1  CDK4  NGFR  HSPA4  HSPA1A  ACTC1  SCN5A  TNFAIP3  PAK2  CREBBP  PDCD6IP  IFI27  STK3  IRF3  KRT18  ATG5  PSEN1  YWHAG  CXCR4  MAP3K1  CSNK2A1  TLR2  KLHL9  IL13  TAFAZZIN  ESR2  TPM1  BCL6  PRPF8  PRF1  NLRP1  CDH1  CTSD  ATR  TSC2  PLK1  KIT  SLC25A4  PDCD10  TNFSF13B  ADA  INS  CREB1  PDCD1  ACTN2  WWOX  STK17B  YWHAH  IL15  BCR  DAPK3  PPIA  MAPT  NOTCH1  H2AX  EZH2  ITPR1  LGALS3  GDNF  EPO  TNNT2  CDC25C  SMPD1  MAP3K7  PRKCE  SPP1  TLR3  DSG2  ALOX5  PRKCI  BMF  IL24  NPM1  FHIT  ERBB3  PTPA  STK11  TNFSF11  RPS3  CADM3  SHC1  CHEK1  RET  STIM1  FOXP3  SPHK1  MFN2  TCN1  FGFR1  ATP1A1  GJA1  GAPDH  CSF2  MADD  PDCD4  LGALS1  CD27  JAK3  LCK  IER3IP1  JAK1  RAC1  CTSB  NUPR1  NTRK1  GSN  GDF15  GSDMD  MKI67  CDKN3  F9  VIM  TOP1  SNCA  RNASEL  MYBPC3  TNFRSF21  FLT3  CAV1  ERN1  ICAM1  ATF2  TRAF6  FAM215A  BAG1  BCAP31  CXCL12  PLEKHF1  TNNI3  EIF5A  PIM1  F2  EIF2AK2  KDR  PKP2  UACA  FGF2  NDRG1  TRAF3  MYH6  TMEM43  IKBKG  CD274  FUT2  LMNB1  IER3  PIDD1  PTPMT1  MT-CYB  DMD  F11  CARD16  SFN  MET  HSP90AB1  ACIN1  YY1  BAG6  PTPN13  TNFRSF12A  TAF6  ACHE  CRADD  CDK6  MDM4  XBP1  NOS3  CCL2  LAMP2  PPM1D  MIR499A  HSPD1  SUMO1  STK24  BNIP3L  BMP6  CD40LG  HGF  CAST  NKX2-5  CSNK2A2  MIR155  STAT5A  ABCC1  PPP1R15A  DYNLL1  JUP  MEF2A  TMBIM6  MAPK11  FGFR3  STYXL1  SREBF1  RPS6KB1  MPZ  BMP4  LRP2  F5  TIMP1  PRKCB  SOCS3  CAPN2  CTLA4  JAG1  CD69  MLH1  MAPK8IP1  MIR34A  CASP8AP2  KAT5  SOS1  FGFR2  MMP2  MT-RNR2  GDAP1  PCSK9  PRKD1  CDK11B  FASN  DAPK2  GORASP1  OPTN  IRS1  ACVR1  S100A9  ATRAID  VWF  DES  NQO1  MYL3 | TP53  ADA  FAS  EGFR  BCL2  PTEN  CASP8  ATM  PIK3CA  AKT1  KRAS  KIT  TNF  PDCD1  CDKN2A  FASLG  CCND1  MYC  BRAF  CTNNB1  CASP3  BAX  ERBB2  STAT3  SCN5A  IL6  MTOR  IFNG  HRAS  TNFRSF10B  CDH1  TGFB1  CD274  NRAS  MAPK1  VHL  FGFR3  RB1  TERT  VEGFA  CD4  IL10  KITLG  ALK  CDKN1A  BAD  LMNA  HLA-DRB1  BCL2L1  BID  BCL6  RIPK1  ABL1  CXCL8  IL2  XIAP  MAPK8  IL1B  ICAM1  TTN  RET  BRCA1  CXCR4  NFKB1  CTLA4  BRCA2  STAT1  JAK2  EPCAM  PTCH1  VCAM1  STK11  JUN  CD44  FLT3  MIR34A  PPARG  ITGB1  SRC  PIK3R1  TNFSF10  NCAM1  EGF  IL4  CD40  C11orf65  MUC1  IL2RA  PDCD1LG2  HIF1A  PTPN11  TGFBR2  CFLAR  BIRC5  NPM1  CDK4  HLA-B  CASP9  HAVCR2  FGF2  PCNA  FH  INS  MCL1  KDR  CYCS  MDM2  NFKBIA  CD40LG  CD28  PARP1  APC  TNFRSF10A  FGFR1  RAC1  WT1  MAPK14  IL7  MITF  VIM  CXCL12  BCR  APP  SYK  IL3  FGFR2  TSC2  ABCB1  NFE2L2  TFRC  IRF1  TRA  KCNH2  CD3E  RELA  FOS  CDK1  CD34  ENG  TNFRSF1A  TP73  PRF1  CAV1  CD247  MMP9  HLA-A  CSF2  SOD1  HMGB1  GZMB  CSF3  FHIT  PTGS2  TSC1  MMP2  TP63  FOXP3  MYD88  TRB  MLH1  CREBBP  KLRK1  GATA2  CALM2  IGF1  TLR4  IGF1R  BTK  IL1A  FN1  CD19  CCL2  ICOS  CD8A  LHCGR  EPO  MAPK3  HGF  THY1  BIRC3  HSP90AA1  SLC4A1  SMARCA4  TET2  CDK6  CD36  ALB  TLR2  MIR20A  POU5F1  MIR29A  GFAP  B2M  CD27  FCGR3A  ITGB2  CREB1  IFNA1  ING1  HMOX1  CEACAM5  CCL5  PDGFRA  IL15  SOX2  ITGA4  FBXW7  MAPT  ITGB3  ANXA5  NGF  CALR  SPP1  GBA  MSH6  PROM1  CSF1  PRKCA  BAK1  IL2RB  CCR5  IL13  IGH  NOS2  ERBB4  VWF  NTRK1  CAT  CLU  CEACAM1  PMS2  ERBB3  CD79A  E2F1  NGFR  SP1  SOX9  CASP1  ENO2  MMP1  ROS1  CEP85L  PLAU  LIG4  DDX3X  MIR19A  MME  IDH1  TNFRSF1B  MS4A1  BIRC2  TLX1  DNMT3A  TGFB2  SELE  MKI67  HLA-G  IL17A  TGFA  ALCAM  CDKN3  SNCA  ICOSLG  TCL1A  THPO  SST  MYCN  AURKA  TLR3  GSK3B  CALM1  THBD  CD80  PDGFB  MIR93  BMP6  HDAC9  BMP2  HSPA5  THBS1  HLA-DQB1  SERPINE1  CHGA  NTRK2  DSE  MPL  IL5  CSF1R  TYR  CASP7  IL18  CD209  FYN  LEP  MPO  CCND2  NR3C1  CTSD  G6PD  TNFSF11  ANK2  IL6ST  PLAUR  CCR6  CD2  KRT19  CR2  ITGAL  BAP1  HSPB1  ABCG2  STAT5A  BSG  GPC3  HSPA4  ABCC1  GAPDH  MMP14  TNFSF13B  TRG  S100B  CD81  F2  EPHA2  CD86  IL21  ITGB4  P2RX7  CEACAM3  PTPN22  PRKDC  MSN  ACE  SELL  CEACAM6  VCP  TJP1  TH  ITGAV  DPP4  ARID1A  ATRX  ITGA6  VDR  HSPD1  SELP  SOD2  NES  CD79B  CCR7  MYLK  NANOG  CACNA1A  GATA4  HSPG2  PARD3  FANCD2  IL12B  CCL3  PSAP  ETS1  FUS  TNFRSF10D  AFP  HLA-C  CD70  TF  MUC4  SHC1  CXADR  TOP2A  EGR1  MBP  MIR27A  CIITA  TNFRSF10C  JAK1  TBK1  BMP4  TNFRSF8  TRAF6  NRG1  CYP1A1  KRT8  NOTCH2  GRP  CD47  ITGAM  CXCR3  CTNNA1  NUMA1  GNAS  BMI1  TIMP1  CD55  NEDD9  ELANE  EPAS1  SCN4A  PDX1  MAP2  F3  PNP  IL4R  CALCA  NFATC2  HLA-DPB1  NFATC1  SEMA3A  BCAP31  FGF1  IRF4  CXCL10  CHAT  PRKCB  CR1  KLF4  PARK7  NLRP3  CD33  ITGA2  BGLAP  PTHLH  PVR  CD46  NTRK3  PLA2G6  ANPEP  ATR  CD69  MIF  HFE  BECN1  FCGR2B  ADAM10  CD38  PDPN  CYP2A6  CA9  LEPR  AHR  LGALS1  NEUROD1  KIR2DL3  CD59  IFNB1  XRCC5  GLB1  TRD  HBG1  IDO1  F5  CP  ITGA2B  TXN  CASP6  CD68  H2AX  HSPA8  CD14  NECTIN1  KIR2DL1  RPS27A  GRN  SMN1  TTR  SERPINA3  MMP3  XRCC6  NT5E  PSCA  BRD4  MYL2  IL11  RIPK3  AREG  COL2A1  VTCN1  TNFRSF11A  CALM3  ELN  FGFR4  CD22  PLAT  C3  TGM2  ALPP  TLR7  MYH9  EIF2AK3  CD99  ANTXR2  ANXA2  VIP  ANXA1  POLE  APOB  AGER  LTA  RHD | ZNF419  ZFP69B  ZFP36  YY1AP1  YWHAE  XBP1  WIPI2  WIPI1  VLDLR  VEGFA  USP7  ULK2  ULK1  UBC  TXNRD1  TXNIP  TUBE1  TTPA  TSC22D3  TRIB3  TP63  TNFAIP3  TMBIM4  TLR4  TIMM9  TGFBR1  TFR2  TFAP2C  TBK1  SUV39H1  STMN1  STK11  STEAP3  SRXN1  SREBF2  SREBF1  SRC  SP1  SOCS1  SNX4  SNORA16A  SMAD7  SLC7A5  SLC39A14  SLC2A8  SLC2A6  SLC2A3  SLC2A14  SLC2A12  SLC2A1  SLC1A4  SLC16A1  SIRT3  SETD1B  SELENOS  SCP2  SAT1  RPTOR  RIPK1  RGS4  RELA  RB1  PVT1  PTGS2  PTEN  PSAT1  PROM2  PRKAA2  PRKAA1  PRDX6  PRDX1  POR  POM121L12  PML  PLIN4  PLIN2  PLA2G6  PIR  PHKG2  PHF21A  PGD  PEX6  PEX3  PEX2  PEX12  PEX10  PEBP1  PCK2  PARK7  PANX1  P4HB  OXSR1  OTUB1  OSBPL9  NT5DC2  NRAS  NR4A1  NR1D2  NR1D1  NQO1  NOX5  NOX3  NOX1  NOS2  NNMT  NGB  NFS1  NF2  NEDD4L  NCF2  MYB  MUC1  MTOR  MTDH  MTCH1  MT3  MT1G  MT1DP  MMP13  MMD  MLLT1  MIR9-3  MIR9-2  MIR9-1  MIR6852  MIR4715  MIR424  miR-378a-3p  MIR30B  MIR214  MIR212  miR-182-5p  MIR17  MIR137  MIOX  MAPK9  MAPK8  MAP3K5  MAP3K11  MAP1LC3A  MAFG  LYRM1  LURAP1L  LRRFIP1  LPIN1  LONP1  LOC390705  LOC284561  LINC00618  LINC00472  LINC00336  LIG3  LCE2C  LAMP2  KRAS  KLHL24  KIM-1  JUN  JDP2  ISCU  INTS2  IL33  IL1B  IFNG  IDH1  HSD17B11  hsa_circ_0008367  HRAS  HNF4A  HILPDA  HIC1  HERPUD1  HELLS  HCAR1  HBA1  HAMP  GSK3B  GRIA3  GPX2  GPT2  GPAT4  GLUT13  GDF15  GCH1  GABPB1  GABARAPL2  GABARAPL1  G6PDX  G6PD  FZD7  FXN  FNDC5  FLT3  FH  Fer1HCH  FBXW7  FBW7  FAR1  FANCD2  FADS2  FADS1  EPT1  EPAS1  ENPP2  EMC2  ELOVL5  EIF2AK4  EGLN2  EGFR  DUSP1  DUOX2  DUOX1  DRD5  DRD4  DPP4  DNAJB6  DECR1  DDIT4  DDIT3  DCAF7  DAZAP1  CYP4F8  CYBB  CYB5R1  CXCL2  CS  CISD2  CISD1  CircIL4R  CHP1  CHMP6  CHMP5  CEBPG  CDO1  CDKN2A  CDH1  CDCA3  CD82  CD44  CAV1  CARS1  CAPG  CA9  BRPF1  BRDT  BRD3  BRD2  BNIP3  BLOC1S5-TXNDC5  BID  BCAT2  BACH1  AURKA  ATP6V1G2  ATM  ATG7  ATG4D  ATG3  ATG16L1  ATG13  ATF2  ASNS  ARRDC3  ARNTL  AQP8  AQP5  AQP3  ANO6  ANGPTL7  AMN  ALOXE3  ALOX15B  ALOX12B  ALDH3A2  ALB  AGPS  AEBP2  ACVR1B  ACSL3  ACOT1  ACO1  ACADSB  ABCC1 | CASP8  BCL2  CASP3  FAS  BAX  XIAP  CFLAR  FASLG  TP53  TNFSF10  CASP9  CASP10  AIFM1  MCL1  BCL2L1  CYCS  TNF  TNFRSF10B  BIRC5  BCL2L11  CASP7  CASP2  PRKCD  BID  TNFRSF10A  PAWR  BOK  AKT1  CASP1  MAPK8  CASP6  BIRC2  PARP1  FADD  DIABLO  SIVA1  BIRC3  RIPK1  KRAS  BAK1  CDKN1A  MOAP1  MAPK1  STAT3  TNFRSF1A  BIRC7  MYC  PEA15  MAP3K5  NRAS  CCAR1  APAF1  MAPK14  ANXA5  BAD  BFAR  AVEN  NOL3  CASP4  BBC3  IL1B  TRAF2  PERP  IL10  TRIAP1  IL6  JUN  TNFRSF10C  TNFRSF10D  IFNG  IL2  MDM2  NFKB1  TRADD  BCL2L2  CASP14  BIK  CXCL8  E2F1  CASP5  MTOR  GZMB  EGFR  SOD2  BCL2L14  HSPA5  IL4  PYCARD  DFFA  PTEN  TGFB1  RAF1  BECN1  GSK3B  TLR4  CDK1  FOXO3  HIF1A  VEGFA  BCL2L10  CD4  DAXX  MIR21  HTRA2  TP73  MAPK3  PTGS2  CDKN2A  ATM  CCND1  TNFSF12  IL2RA  PMAIP1  RELA  TNFRSF25  MAPK10  IGF1R  F10  TET2  IGF1  IL15  JAK2  RNF34  CLU  SIRT1  PTK2  MAPK9  IL1A  PIK3CG  PML  IL18  CDK2  HDAC9  ICAM1  DAPK1  NFKBIA  FOS  TLR2  BAG3  IL3  VDAC1  CTLA4  BCL2A1  TXN  SRC  RIPK3  PPP1R13B  DFFB  CXCR3  TNFRSF1B  TRAF1  IL5  BIRC6  CCL2  DDIT3  APP  FAF1  BRCA1  IFI27  PPARG  STK4  BCL10  ATG5  HMGB1  MAP2K1  ABL1  CDKN1B  NGF  CTNNB1  RB1  HSPB1  HMOX1  ERBB3  CD40  TERT  AR  ESR1  EGF  NR4A1  STAT1  TNFRSF11B  TNFAIP3  SOD1  IFNA1  CHUK  F3  NFE2L2  CAT  PIK3CA  YAP1  PAK2  KRT18  HRAS  ERBB2  MPO  CHEK2  TP63  NR3C1  PCNA  CD274  RARA  TLR3  IKBKB  YWHAB  ALB  ENDOG  HSPA4  MADD  HSP90AA1  CCNB1  ABCB1  CCNA2  IFNB1  IRF1  PRPF8  HIPK2  MMP9  NOS2  CCL5  SQSTM1  NLRP1  YWHAQ  CDK4  CD69  DAPK3  IRF3  TNFRSF12A  F7  MAP2K4  FCER2  NGFR  HSPA1A  PRKCA  CDK5  PIK3R1  CD27  WWOX  PRF1  PIDD1  PTPA  IL13  GSK3A  YWHAE  ELANE  IGHE  CXCR4  SMPD1  FOXO1  MAP3K7  SP1  PDCD1  IL24  CDH1  PRKN  CD8A  IL17A  TFPI  ATR  STK11  EPO  KIT  BDNF  CSF2  IGFBP3  TNFSF11  HGF  BNIP3  CASP12  STK3  CAV1  CCL3  DNM1L  NPM1  H2AX  F2  CRADD  SELL  YWHAZ  C1QBP  TNFSF13B  FN1  CXCL2  FLT3  SFTPD  FAM215A  FAIM2  PIM1  CHEK1  PLK1  ITPR1  LGALS3  SHC1  SFRP1  STAT6  UNC5B  IER3  PRKCI  EP300  BCAP31  PTPN13  GSN  NOTCH1  EZH2  DNASE1L3  CRP  JAK1  YWHAG  ESR2  RPS6KB1  VHL  GAPDH  YWHAH  CCR6  BRAF  MMP2  CDK6  RFFL  TNFRSF21  CD40LG  NOS3  DEDD  TNNI3  CASP8AP2  ACIN1  FGF2  BMP6  PTPRC  DNAJA3  DAPK2  HLA-DRB1  LCK  LMNA  PCSK9  SPP1  GDF15  F5  IL9  MIR146A  ITGAL  INS  PTPN11  BMP4  TOP1  SERPINC1  HP  NTRK1  TIMP3  ALOX5  ULK1  GADD45A  ANXA1  MAP3K14  MET  BCL2L12  CREB1  MIR210  FGFR3  ODC1  LYN  TMBIM4  KITLG  BEX3  FOXP3  KAT5  OLR1  RET  ITGAM  PI3  VIM  CDC25C  RAC1  BCL2L2-PABPN1  PLG  MIR16-1  BAG6  GCG  UNC5A  ABCC1  PRKCZ  ZC3H12A  VCAM1  IFIH1  LTBR  NFKB2  EGR1  EIF2S1  FGFR2  DUSP19  THBS1  BMP2  SOCS3  FANCD2  ARFGEF2  PTK2B  MKI67  MLKL  ACHE  CSF3  XBP1  TIMP1  PDPK1  CXCL12  CCND3  IL7  SUMO1  FASN  PSEN1  MAPT  FAIM  NDUFS4  HSP90AB1  NLRP3  MIR223  FCMR  MEF2A  MIR590  F9  EIF2AK3  MIR142  SYK  MYCN  TGFBR1  HSPA8  TGFA  SIAH1  NUMA1  IRS1  STAT5B  ACE  PLAUR  RHOA  TMBIM1  SMAD7  FGFR1  NOX4  MALT1  CD80  MIR155  DEDD2  BCL2L15  DDX3X  GDNF  PAK1  PRDX2  LCN2  SFPQ  SFRP2  SMPD2  EDN1  ITK  CYLD  GSTP1  NTRK2  TRAF5  SERPINE1  ITGAV  CXCL5  IFI6  TNFRSF8  CD36  P2RX7  HTT  AGER  SMAD2  NRG1  CBL  S100A9  SPTAN1  PROC  CCR5  ATG14  LEP  PRKCQ  F2R  NRP1  SKP2  PHLPP1  SET  PIK3C2A  DAB2IP  MIR199A1  GADD45B  IGF2  APEX1  MYH6  IAPP  IL4R  MIR19B1  PRKAA1  PRNP  ITGB1  PRKDC  NOS1  RB1CC1  ROCK1  G0S2  HMGCR  PPARA  CREBBP  CAPN1 | TP53  MTOR  TNFSF10  GZMB  EZR  MRTFA  DIAPH1  MAP1LC3A  LPAR2  CDH1  CTNNA1  AMPK  RHOA  ROCK  ATG5  ATG7  PI3KC3  BECN1  CYBB  UVRAG  RUBCN  CDC42  RNF146 | ATP7A  ATP7B  CDKN2A  DBT  DLAT  DLD  DLST  FDX1  GCSH  GLS  LIAS  LIPT1  MTF1  PDHA1  PDHB  SLC31A1  SLC25A3 | AMBRA1  APOL1  ARNT  ARSA  ARSB  ATF4  ATF6  ATG10  ATG12  ATG16L1  ATG16L2  ATG2A  ATG2B  ATG3  ATG4A  ATG4B  ATG4C  ATG4D  ATG5  ATG7  ATG9A  ATG9B  ATIC  BAG1  BAG3  BAK1  BAX  BCL2  BCL2L1  BECN1  BID  BIRC5  BIRC6  BNIP1  BNIP3  BNIP3L  C12orf44  C17orf88  CALCOCO2  CAMKK2  CANX  CAPN1  CAPN10  CAPN2  CAPNS1  CASP1  CASP3  CASP4  CASP8  CCL2  CCR2  CD46  CDKN1A  CDKN1B  CDKN2A  CFLAR  CHMP2B  CHMP4B  CLN3  CTSB  CTSD  CTSL1  CX3CL1  CXCR4  DAPK1  DAPK2  DDIT3  DIRAS3  DLC1  DNAJB1  DNAJB9  DRAM1  EDEM1  EEF2  EEF2K  EGFR  EIF2AK2  EIF2AK3  EIF2S1  EIF4EBP1  EIF4G1  ERBB2  ERN1  ERO1L  FADD  FAM48A  FAS  FKBP1A  FKBP1B  FOS  FOXO1  FOXO3  HDAC1  HDAC6  HGS  HIF1A  HSP90AB1  HSPA5  HSPA8  HSPB8  IFNG  IKBKB  IKBKE  IL24  IRGM  ITGA3  ITGA6  ITGB1  ITGB4  ITPR1  GAA  GABARAP  GABARAPL1  GABARAPL2  GAPDH  GNAI3  GNB2L1  GOPC  GRID1  GRID2  KIAA0226  KIAA0652  KIAA0831  KIF5B  KLHL24  LAMP1  LAMP2  MAP1LC3A  MAP1LC3B  MAP1LC3C  MAP2K7  MAPK1  MAPK3  MAPK8  MAPK8IP1  MAPK9  MBTPS2  MLST8  MTMR14  MTOR  MYC  NAF1  NAMPT  NBR1  NCKAP1  NFE2L2  NFKB1  NKX2-3  NLRC4  NPC1  NRG1  NRG2  NRG3  P4HB  PARK2  PARP1  PEA15  PELP1  PEX14  PEX3  PIK3C3  PIK3R4  PINK1  PPP1R15A  PRKAB1  PRKAR1A  PRKCD  PRKCQ  PTEN  PTK6  RAB11A  RAB1A  RAB24  RAB33B  RAB5A  RAB7A  RAC1  RAF1  RB1  RB1CC1  RELA  RGS19  RHEB  RPS6KB1  RPTOR  SAR1A  SERPINA1  SESN2  SH3GLB1  SIRT1  SIRT2  SPHK1  SPNS1  SQSTM1  ST13  STK11  TBK1  TM9SF1  TMEM49  TMEM74  TNFSF10  TP53  TP53INP2  TP63  TP73  TSC1  TSC2  TUSC1  ULK1  ULK2  ULK3  USP10  UVRAG  VAMP3  VAMP7  VEGFA  WDFY3  WDR45  WDR45L  WIPI1  WIPI2  ZFYVE1 | TP53  TERT  PTCH1  MYC  VIM  BCL2  MAPK1  TNF  DNAH8  EGFR  FAS  GSN  RAF1  CASP3  CCND1  JAK2  AR  TUBB  NDE1  GLI1  CYCS  SALL4  MDM2  AIFM1  CAV1  EDN1  MKI67  SNCA  CDKN1A  CD40  EDNRB  BAX  NOS2  APP  PRKCD  USP9X  TREX1  ATRIP  TGM1  ETS1  NOS3  CASP9  CD4  MAP3K1  PARP1  LEP  ANXA5  JUN  SLC6A3  IL3  CD44  THBS1  CUL3  NME1  BCL2L1  CASP7  PRKCA  EPO  SIRT1  FDFT1  CAT  TLR3  BIRC5  REN  MCL1  JAK3  MAPK8  NOS1  PRKCE  BIRC8  ADIPOQ  ANXA1  HTRA2  FDPS  LTF  CASP6  CASP1  DNASE1  CD47  TNFSF10  RASSF1  XDH  CHP1  TUBA4A  CASP2  CAPN1  TP73  LALBA  MAPK14  F2R  GADD45A  STAT5A  APOL1  FAM111A  DYNC1I2  ATRIP-TREX1  BAK1  PLA2G4A  TNFRSF10A  TNFRSF10B  APAF1  BECN1  TERF1  BCL2L11  VDAC1  SIRT3  TUBA1B  LINGO1  IDO1  STK17B  HERPUD1  TFPT  RUNX3  SIRT2  ENDOG  BAD  ATP12A  PDE5A  PSIP1  ATOH1  TOP3B  MIR130A  PPP3R2  PRDX6  PRDX5  PDGFD  SQLE  H2BC21  H1-1  GZMA  GPR87  BCL2L2  RPS5  UBE3C  PRDX4  MAPK13  WNK3  ADRB3  BIK  H3C15  PLAAT4  BCL2L10  TDRD3  SERPINB9  ANKRD36B  MIOX | CEACAM5  PTK2  PIK3CA  CEACAM6  PDK4  BCL2  TSC2  PTRH2  STK11  MAP3K7  NTRK2  CRYBA1  MCL1  DAPK2  NOTCH1  TLE1  CAV1  SNAI2  IKBKG  SRC  AKT1  MTOR  CHEK2  E2F1  ITGA5  ITGB1  BRMS1 |

Supplementary Table 2. Other models had been established for ovarian cancer.

| Signature | Title | PMID |
| --- | --- | --- |
| An  Any  Bao  Bi  Bing  Cao  Chaofan  Chen  Cheng  Ding  Fan  Fang  Fei  He  Hu  Huan  Huo  Jiao  Jin  JinC  Jinwei  Khadirnai  kar  Lei  Leilei  Li  Liang  Lin  Liu  Lixiao  Na  Nie  Pan  PanX  Peng  Qi  Qingyang  Qiu  Sheng  Su  Sun  Wang  Xiang  Xiaoqing  Xin  Xu  Xueyuan  Xuyao  Yan  Yilong  Yang  Yuanyuan  Zeng  Zhao | Development of a Novel Autophagy-related Prognostic Signature for Serous Ovarian Cancer  The Comprehensive Analysis of Interferon-Related Prognostic Signature with regard to Immune Features in Ovarian Cancer.  Novel gene signatures for prognosis prediction in ovarian cancer  Establishment of a novel glycolysis-related prognostic gene signature for ovarian cancer and its relationships with immune infiltration of the tumor microenvironment  Novel Model for Comprehensive Assessment of Robust Prognostic Gene Signature in Ovarian Cancer Across Different Independent Datasets  Development of a multi-gene-based immune prognostic signature in ovarian Cancer  Establishment and validation of an RNA binding protein-associated prognostic model for ovarian cancer  Integrating cell cycle score for precise risk stratification in ovarian cancer  Construction and validation of a transcription factors-based prognostic signature for ovarian cancer.  Construction of a new tumor immunity-related signature to assess and classify the prognostic risk of ovarian cancer  A newly defined risk signature, consisting of three m6A RNA methylation regulators, predicts the prognosis of ovarian cancer  Establishment, immunological analysis, and drug prediction of a prognostic signature of ovarian cancer related to histone acetylation  Construction autophagy-related prognostic risk signature to facilitate survival prediction, individual treatment and biomarker excavation of epithelial ovarian cancer patients.  Development of a novel transcription factors-related prognostic signature for serous ovarian cancer  Identification of a five-gene signature of the RGS gene family with prognostic value in ovarian cancer.  Integrated Analysis of Ferroptosis-Related Biomarker Signatures to Improve the Diagnosis and Prognosis Prediction of Ovarian Cancer  Identification of a Prognostic Signature for Ovarian Cancer Based on the Microenvironment Genes  N6-Methyladenosine-Related RNA Signature Predicting the Prognosis of Ovarian Cancer.  A panel of three oxidative stress-related genes predicts overall survival in ovarian cancer patients received platinum-based chemotherapy  A 2-Protein Signature Predicting Clinical Outcome in High-Grade Serous Ovarian Cancer  Identification and verification of a ten-gene signature predicting overall survival for ovarian cancer  Development and validation of an immune prognostic signature for ovarian carcinoma  Identification of an energy metabolism‑related gene signature in ovarian cancer prognosis  Establishment and validation of a novel invasion-related gene signature for predicting the prognosis of ovarian cancer.  Identification and validation of a gene-based signature reveals SLC25A10 as a novel prognostic indicator for patients with ovarian cancer  A Novel Glycosyltransferase-Related Gene Signature for Overall Survival Prediction in Patients with Ovarian Cancer  A methylation-driven genes prognostic signature and the immune microenvironment in epithelial ovarian cancer.  Construction and validation of a novel aging-related gene signature and prognostic nomogram for predicting the overall survival in ovarian cancer.  Construction and Validation of a Novel Glycometabolism-Related Gene Signature Predicting Survival in Patients With Ovarian Cancer.  Comprehensive Analysis of Tumor Microenvironment Identified Prognostic Immune-Related Gene Signature in Ovarian Cancer  Prognostic signature of ovarian cancer based on 14 tumor microenvironment-related genes  A Potential Immune-Related Long Non-coding RNA Prognostic Signature for Ovarian Cancer  A Novel Six-Gene Signature for Prognosis Prediction in Ovarian Cancer  A prognostic model based on immune-related long noncoding RNAs for patients with epithelial ovarian cancer  A nine-gene signature related to tumor microenvironment predicts overall survival with ovarian cancer  Identifying the Role of Oxidative Stress-Related Genes as Prognostic Biomarkers and Predicting the Response of Immunotherapy and Chemotherapy in Ovarian Cancer.  A Liquid-Liquid Phase Separation-Related Gene Signature as Prognostic Biomarker for Epithelial Ovarian Cancer  Integrative network analysis identifies an immune-based prognostic signature as the determinant for the mesenchymal subtype in epithelial ovarian cancer.  A novel immune-related prognostic signature in epithelial ovarian carcinoma  Identification of a Prognostic Signature Associated With DNA Repair Genes in Ovarian Cancer.  Development of a five-gene signature as a novel prognostic marker in ovarian cancer.  Construction of a prognostic signature for serous ovarian cancer based on lactate metabolism-related genes.  Identification of immunity- and ferroptosis-related genes for predicting the prognosis of serous ovarian cancer  The cuproptosis-related gene signature serves as a potential prognostic predictor for ovarian cancer using bioinformatics analysis  Integration of Transcriptome and Epigenome to Identify and Develop Prognostic Markers for Ovarian Cancer  Derivation, Comprehensive Analysis, and Assay Validation of a Pyroptosis-Related lncRNA Prognostic Signature in Patients With Ovarian Cancer  A signature based on glycosyltransferase genes provides a promising tool for the prediction of prognosis and immunotherapy responsiveness in ovarian cancer  Development and Verification of an Autophagy-Related lncRNA Signature to Predict Clinical Outcomes and Therapeutic Responses in Ovarian Cancer  Identification of a novel ferroptosis-related gene signature associated with prognosis, the immune landscape, and biomarkers for immunotherapy in ovarian cancer  Integrated analysis of a competing endogenous RNA network reveals an 11-lncRNA prognostic signature in ovarian cancer  Development and Validation of an Immune-Related Prognostic Signature for Ovarian Cancer Based on Weighted Gene Coexpression Network Analysis  Identification of a Gene Signature of Cancer-Associated Fibroblasts to Predict Prognosis in Ovarian Cancer  Exploration of the Immunotyping Landscape and Immune Infiltration-Related Prognostic Markers in Ovarian Cancer Patients.  Identification three LncRNA prognostic signature of ovarian cancer based on genome-wide copy number variation | 30410611  35769811  32666642  34496868  31681404  33509250  33550985  36061171  35227285  33154188  32950970  36172179  33676525  33785763  33845140  35071242  34054929  34137363  29910195  28976449  32805252  32794637  32186777  35292033  36114504  34992448  35783253  34825509  33281878  33679883  34260536  34367253  33193589  35031063  32208363  36561981  34168991  33031300  33819196  31572446  30569721  36185201  35777713  36267774  36081667  35280739  36611197  34671615  36386203  33223503  33381581  35873482  35880167 |
