# Supplementary figures and images for: Machine Learning Developed a Programmed Cell Death Signature for Predicting Prognosis, Ecosystem, and Drug Sensitivity in Ovarian Cancer
Source: Anal Cell Pathol (Amst). 2023 Oct 11;2023:7365503. doi: 10.1155/2023/7365503 (PMC10586435; doi:10.1155/2023/7365503)

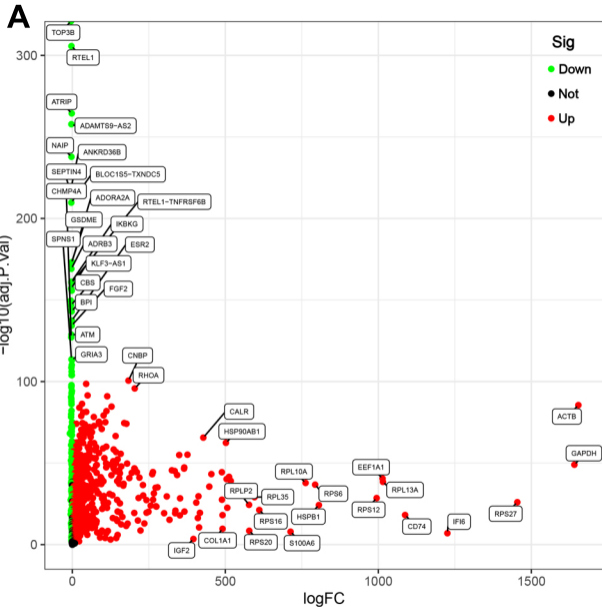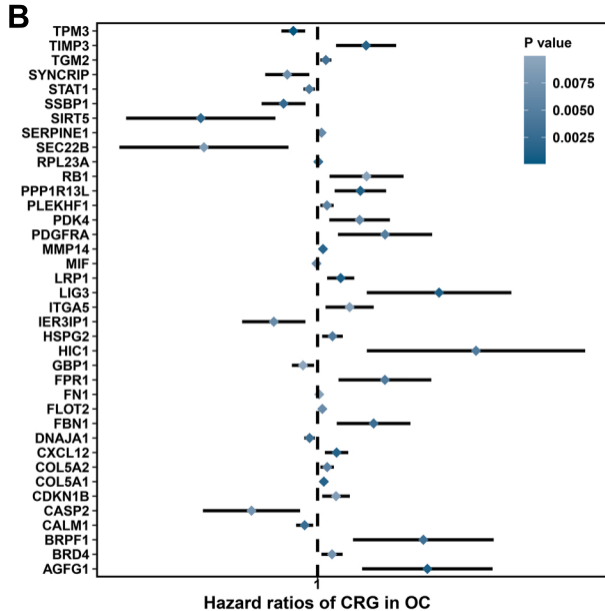

Supplement: Supplementary 3 — Potential biomarkers among PCD-related genes in ovarian cancer. [file 7365503.f3.pdf]

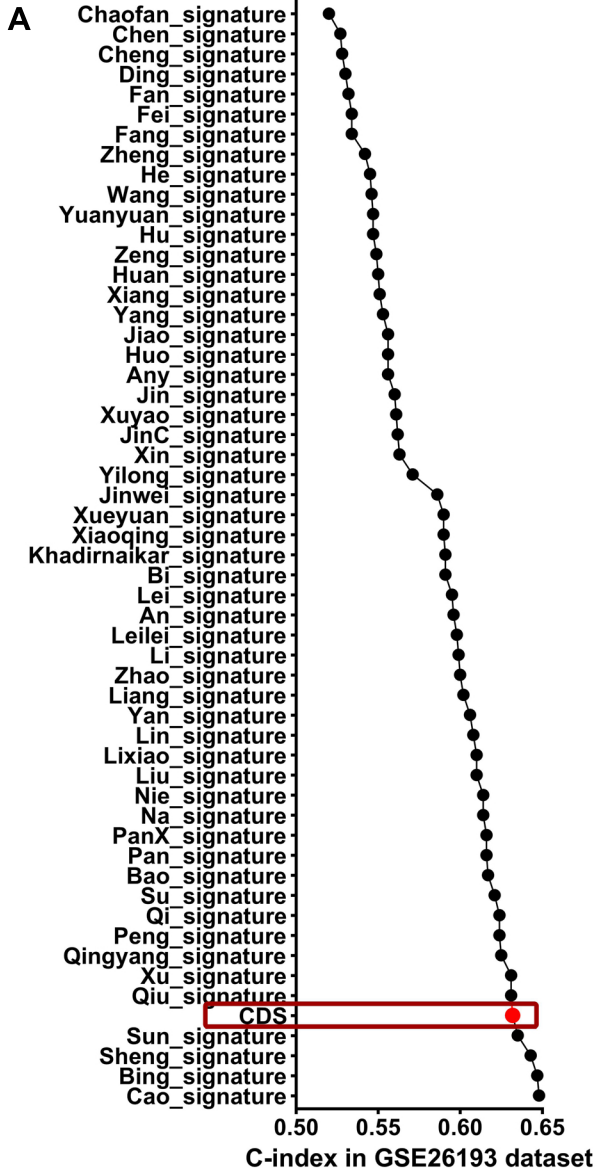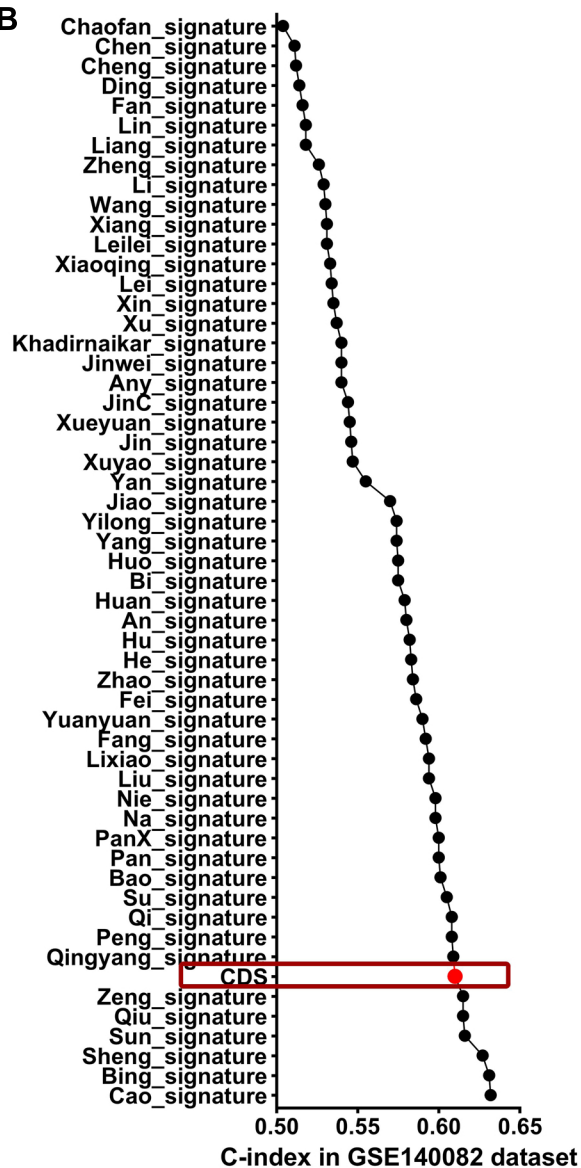

Supplement: Supplementary 4 — Evaluation of the performance of CDS in predicting the clinical outcome of OC patients. [file 7365503.f4.pdf]
